# Arginine in *C9ORF72* Dipolypeptides Mediates Promiscuous Proteome Binding and Multiple Modes of Toxicity*[Fn FN2]

**DOI:** 10.1074/mcp.RA119.001888

**Published:** 2020-02-21

**Authors:** Mona Radwan, Ching-Seng Ang, Angelique R. Ormsby, Dezerae Cox, James C. Daly, Gavin E. Reid, Danny M. Hatters

**Affiliations:** ‡ Department of Biochemistry and Molecular Biology; and Bio21 Molecular Science and Biotechnology Institute, The University of Melbourne, VIC 3010, Australia; § Bio21 Mass Spectrometry and Proteomics Facility, The University of Melbourne, Parkville, Victoria, Australia; ¶ School of Chemistry, The University of Melbourne, VIC 3010, Australia

**Keywords:** Neurodegenerative diseases, protein-protein interactions, methylation, translation, protein identification, molecular biology, networks, amyotrophic lateral sclerosis (ALS), methylosome, protein aggregation, proteotoxicity, RAN-translation

## Abstract

*C9ORF72*-associated Motor Neuron Disease patients feature abnormal expression of 5 dipeptide repeat (DPR) polymers. Here we used quantitative proteomics in a mouse neuronal-like cell line (Neuro2a) to demonstrate that the Arg residues in the most toxic DPRS, PR and GR, leads to a promiscuous binding to the proteome compared with a relative sparse binding of the more inert AP and GA. Notable targets included ribosomal proteins, translation initiation factors and translation elongation factors. PR and GR comprising more than 10 repeats appeared to robustly stall on ribosomes during translation suggesting Arg-rich peptide domains can electrostatically jam the ribosome exit tunnel during synthesis. Poly-GR also recruited arginine methylases, induced hypomethylation of endogenous proteins, and induced a profound destabilization of the actin cytoskeleton. Our findings point to arginine in GR and PR polymers as multivalent toxins to translation as well as arginine methylation that may explain the dysfunction of biological processes including ribosome biogenesis, mRNA splicing and cytoskeleton assembly.

## Abstract (precis)

C9ORF72-associated Motor Neuron Disease patients feature abnormal expression of 5 dipeptide repeat (DPR) polymers. We found the most toxic DPRs, PR and GR, were particularly promiscuous binders to endogenous proteins. This included ribosomal proteins, translation initiation factors and translation elongation factors. The corresponding biological impacts were multipronged and included stalling of ribosomes during translation, hypomethylation of endogenous proteins, and the destabilization of the actin cytoskeleton. The findings point to new mechanisms of toxicity in disease caused by arg-rich DPRs.

## Graphical Abstract

**Figure:**
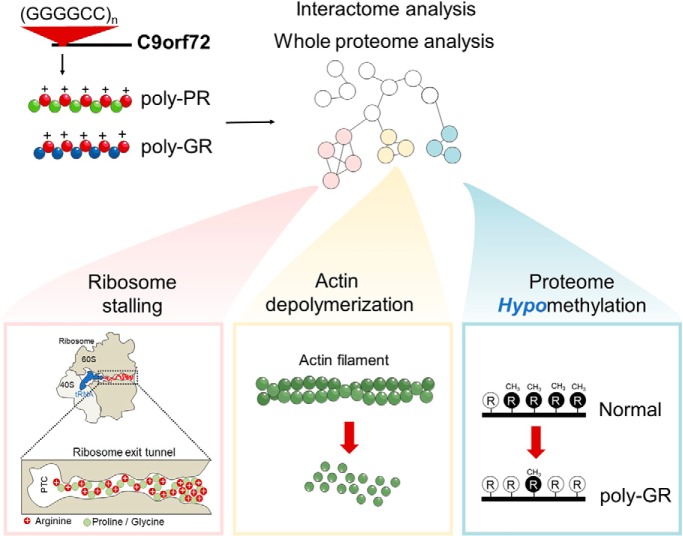

**Highlights**

- Quantitative proteome interactions with 5 different *C9ORF72* dipolypeptides (DPRs).
- The arg-rich DPRs promiscuously bound to the proteome compared with the other DPRs.
- Long repeat lengths of arg-rich DPRs, but not short lengths, stalled ribosomes.
- The arg-rich DPRs also reduced arginine methylation and actin cytoskeleton assembly.

The major genetic cause of motor neuron disease (MND)^1^ (also known as amyotrophic lateral sclerosis (ALS)) and frontotemporal dementia (FTD) is an expansion in the number of GGGGCC hexanucleotide repeats in *C9ORF72* from less than 15 in the general population to over 20 (and typically hundreds) (1–4). Toxicity has been proposed to arise through multiple mechanisms including *C9ORF72* haploinsufficiency (2), the formation of *C9ORF72* mRNA foci that sequesters critical RNA binding proteins (5–10) and the production of abnormal translation products by non-AUG-initiated translation (RAN translation) (11). RAN translation occurs from expanded hexanucleotide repeat lengths in both sense and antisense transcripts resulting in abnormal expression of 5 dipeptide repeats (DPRs) in neurons of patient brains: poly-GP, poly-GA, poly-GR, poly-AP and poly-PR (5, 12–14).

Experimental animal and cell culture models expressing the DPRs have revealed poly-GR and poly-PR to be particularly toxic, with the others being comparatively inert (15–18). Furthermore, although all DPRs are widely distributed in human brain of patients with ALS, only poly-GR is correlated to clinically related regions (19). Various interactome studies have indicated that the poly-GR and poly-PR DPRs engage with RNA binding proteins, ribosome machinery and proteins with low complexity domains, which mediate the formation of membrane-less organelles by phase separation (18, 20, 21). These interactions have been proposed to negatively impact on the functioning of ribosome biogenesis (22), ribosome activity (23), nucleolus function (22, 24), nucleocytoplasmic transport (25, 26) and stress granule dynamics (18, 20, 24).

Here we sought to probe the role of the poly-GR and poly-PR DPRs expressed in a simple cell model by defining what they interact with using quantitative proteomics and examining how short DPR lengths (10× repeats) differed to longer lengths (101× repeats). Our proteomics data suggested potent engagement of poly-PR and poly-GR to ribosome and translational machinery. Given that poly-GR and poly-PR suppress protein translation, we sought to explore the role of the interactions of ribosomes further (18, 20, 21, 23). Here we provide evidence that disruption of protein translation may arise by Arg-rich peptides stalling on ribosomes during their synthesis. We also reveal other key mechanisms mediating poly-PR and poly-GR toxicity. This includes destabilization of the actin cytoskeleton and proteome arginine hypomethylation. Our findings point to the repetitive arginine sequences in the DPRs promoting promiscuous binding to the proteome that in turn enact multiple modes of toxicity.

## EXPERIMENTAL PROCEDURES

### 

#### 

##### Experimental Design and Statistical Rationale

The current study was designed with a focus on identifying how expression of the DPRs altered the abundance of the proteome and to define which proteins engaged to the DPRs. We also were interested in how DPR length affected these properties. We undertook this design by using GFP tagged forms to understand the abundances of the DPRs in the cells and as a handle to capture interactors using GFP trap. The general design was to perform biological replicates for statistical quantitation (between 3 and 4 depending on the sample, which was deemed enough based on expectation that substantial changes in the proteome would arise) as matched pairs with a GFP construct *versus* a DPR-fusion. These constructs were transfected in cells. We compared GFP alone, with GFP fusions of 10× DPR repeats and 101× repeats and used 3-way dimethyl labeling to enable quantitative comparisons between these treatments. The details of the statistical tests that we performed to compare the differences are included in detail in the results section and the figures.

##### Plasmids

Synthetic genes for short (10×) and (101×) dipeptide repeats were synthesized by GeneArt (Life Technologies, Regensburg, Germany). The full sequence information is available (supplemental Table S1). These constructs were flanked 5′ by XhoI and PstI recognition sites and 3′ by BclI and BamHI recognition sites. XhoI and BamHI enzymes (NEB, Ipswich, Massachusetts) were used to introduce the gene cassette into the mammalian expression vector pEGFP-C2 vector so the GFP tag was on the N terminus of the 10× repeat sequence. The 101× DPR sequence were inserted between PstI and BclI sites in the backbones of the previously developed pEGFP-C2–10× DPR via classical restriction digest cloning following manufacturers' recommendations. Transformations were performed using recombination-deficient Stbl3 *E. coli* (Life Technologies) at 30 °C.

##### Cell Culture

Neuro-2a and HEK293T cells, obtained originally from the American Type Culture Collection (ATCC, Manassas, Virginia), were maintained in Opti-MEM (Life Technologies) and Dulbecco's modified Eagle medium (DMEM) (Life Technologies), respectively. The medium was supplemented with 10% v/v fetal calf serum, 1 mm glutamine, and 100 Unit ml^−1^ penicillin and 100 μg/ml streptomycin, and cells were kept in a humidified incubator with 5% v/v atmospheric CO_2_ at 37 °C.

##### Flow Cytometry

For analysis of DPR expression levels, cells were harvested 48 h post-transfection, in 500 μl PBS containing 0.5 μl of 5 μm SYTOX Red dead cell stain (Invitrogen). Cells were analyzed using LSRFortessa X-20 flow cytometer (BD Biosciences). 120,000 events per sample were collected at a high flow rate. Side and forward scatter height, width, and area were collected to gate for single live cell population. GFP flouresence were collected with the 488-nm laser and FITC (530/30) filter to gate for transfected cells. For SYTOX Red dead cell stain, fluorescence was collected using 640-nm laser and APC (670/14) filter. Flow cytometric gating and data analysis was performed using FlowJo software (v10.5.3) and graphs were analyzed in GraphPad Prism 7.05.

##### Confocal Imaging

Cells expressing GFP-tagged DPRs were fixed 48 h after transfection in 4% paraformaldehyde for 15 min at room temperature. Nuclei were counterstained with Hoechst 33342 at 1:200 dilution (Thermo Fisher Scientific, San Jose, CA) for 30 min then washed twice in PBS. Fixed cells were imaged on a Leica SP5 confocal microscope using HCX PL APO CS 40× or 63× oil-immersion objective lens (NA 1.4) at room temperature. The Hoechst 33342 channel was collected with an excitation wavelength of 405 nm and emission wavelengths of 445–500 nm; EGFP was collected by excitation at 488 nm and emission from 520–570 nm. Single color controls were used to establish and adjust to remove bleed through of the emission filter bandwidths. FIJI version of ImageJ (27) and Inkscape were used for image processing.

##### Longitudinal Live Cell Imaging

Neuro-2a cells in 12-well plate format were co-transfected with individual GFP-tagged DPRs along with mCherry in a pT-Rex vector (Life Technologies). The media was refreshed 24 h after transfection and cells were then imaged longitudinally with a JuLI stage live cell imaging system with fluorescent images acquired at 15 min intervals for 96 h (Nanoentek, Seoul, South Korea). Channels used: GFP for EGFP (Excitation: 466/40, Emission: 525/50), RFP for mCherry (Excitation: 525/50, Emission: 580 LP).

Death was recorded as the time points at which mCherry fluorescence was lost. This event corresponded to the loss of membrane integrity and cell death and was found to be a highly sensitive and specific assay of cell death through different pathways and in different types of cell (28, 29). Cells that drifted from focus were censored. For statistical analysis, survival time was defined as the imaging time point at which a cell was last seen alive. Kaplan-Meier curves were used to estimate survival and hazard functions with GraphPad Prism software. Differences in Kaplan-Meier curves were assessed with Log-rank (Mantel-Cox) test.

##### Sample Preparation for Proteome Analysis of GFP-immunoprecipitated Samples

6 × 10^6^ Neuro-2a cells were seeded into 75 cm^2^ flasks and transfected the following day with either GFP-tagged DPRs or GFP-only constructs (24 μg DNA and 60 μl Lipofectamine 2000) according to the manufacturer's instructions (Life Technologies). The experiment was designed as 3 or 4 biological replicates. Media was refreshed 24h after transfection. At 48 h post-transfection, cells were gently rinsed with PBS and harvested in PBS by gently pipetting. Cells were pelleted (120 *g*; 6 min; room temperature) and resuspended in 1 ml PBS and pelleted again (400 × *g*; 6 min; room temperature). The pellet was resuspended in 200 μl ice-cold lysis buffer (10 mm Tris-HCl, pH 7.4; 150 mm NaCl; 0.5 mm EDTA; 0.5% v/v NP-40; 1 mm PMSF; 10 units/ml DNase I) supplemented with EDTA-free Complete protease inhibitor mixture (Roche Diagnostic, Basel, Switzerland). The cell suspensions were then passed through a 27 Gauge syringe needle 25 times, followed by a 31 Gauge needle 10 times and incubated on ice for 30 min. The resultant lysates were clarified by centrifugation (21,000 × *g*; 10 min; 4 °C). Protein concentrations were quantified by the Pierce BCA Protein Assay (Catalogue Number: 23225, Thermo Fischer Scientific, MA) using bovine serum albumin (BSA) as the mass standard. 0.5 mg of cellular protein was added to 25 μl of GFP-Trap MA beads (ChromoTek) pre-washed and equilibrated in the wash buffer (10 mm Tris/Cl pH 7.4; 150 mm NaCl; 0.5 mm EDTA; 1 mm PMSF; EDTA-free Protease inhibitor mixture). The solution was incubated for 2 h at 4 °C (end-over-end rotation). Magnetically separated beads were then washed 3 times with wash buffer and 2 times more with 25 mm triethylammonium bicarbonate (TEAB) buffer. The immunoprecipitated proteins were eluted by the addition of 100 μl of 50% v/v aqueous 2,2,2-Trifluoroethanol (TFE), 25 mm TEAB. The supernatant was collected after pelleting (2000 × *g*; 2 min; room temperature) and adjusted to a final concentration of 100 mm TEAB by addition of 1 m stock solution (and the pH was validated to be ∼7 after this treatment). The samples were further processed for mass spectrometry analysis.

##### Sample Preparation for Whole Proteome Analysis

Neuro2a cells expressing GFP-tagged 101× DPRs were harvested 48 h post transfection in PBS with a cell scraper and gentle pipetting. Cells were pelleted (120 × *g*; 6 min) and resuspended in 2 ml PBS supplemented with 10 units/ml DNase I and filtered through 100-μm nylon mesh before analysis by flow cytometry. Just before sorting, 2 μl of the nuclear marker DAPI (1:1000, D1306, Thermo Fisher Scientific) was spiked into cell suspensions to stain dead cells. Cells were sorted using a FACS ARIA III cell sorter (BD Biosciences) equipped with 405-nm, 488-nm, 561-nm and 640-nm lasers using a 100-μm nozzle. Gating was performed with BD FACS Diva software (BD Biosciences). Cells (1,000,000) of each population of interest were sorted at a speed of 1500 cells/s. Side scatter (SSC) and forward scatter (FSC) height, width, and area were collected to gate for the single cell population. DAPI area was collected to gate-out dead cells. Data were also collected for pulse height, width, and area of GFP with the FITC filter. To match for expression, cells were further gated to the same median GFP intensity of 2200 fluorescence units by varying the window of expression. Cells were sorted in parallel across 3 days and performed as three matched replicates. Cells were kept on ice for all steps of the sorting preparation and handling. The targeted population was directly sorted into PBS, pelleted (120 × *g*, 6 min), and snap frozen in liquid nitrogen then kept at −80 °C until use.

Cell pellets were thawed and resuspended in 100 μl RIPA lysis buffer (25 mm Tris-HCl, pH 7.4, 150 mm NaCl, 1% NP40, 0.1% SDS, 1% Sodium deoxycholate, 1× complete mini-protease mixture; Roche), and incubated on ice for 30 min. The concentration of proteins was measured by the Pierce BCA Protein Assay according to the manufacturer's instruction (Thermo Fisher Scientific). Equal amounts of protein for each sample were precipitated with six volumes of pre-chilled (−20 °C) acetone, and incubation overnight. Samples were pelleted at 21,000 × *g* at 4 °C for 10 min. Acetone was decanted without disturbing the protein pellet. The pellets were washed once with pre-chilled acetone then allowed to dry for 10 min. The protein precipitates were resuspended in 100 μl 0.1 m TEAB and were vortexed and sonicated 3 times for 30 s to help solubilize the pellet. The samples were further processed for mass spectrometry analysis.

##### Mass Spectrometry Analysis

Proteins were subjected to reduction with 10 mm tris(2-carboxyethyl)phosphine (TCEP), pH 8.0, and alkylation with 55 mm iodoacetamide for 45 min, followed by trypsin digestion (0.25 μg, 37 °C, overnight). The resultant peptides were then desalted by solid-phase extraction following acidification in 1% v/v formic acid; the cartridge (Oasis HLB 1 cc Vac Cartridge, product number 186000383, Waters Corp., Milford, Massachusetts) was pre-washed with 1 ml of 80% v/v acetonitrile (ACN) containing 0.1% v/v trifluoroacetic acid (TFA) and equilibrated with 1.2 ml of 0.1% v/v TFA three times. Samples were then loaded on the cartridge and washed with 1.5 ml of 0.1% v/v TFA before being eluted with 0.8 ml of 80% v/v ACN containing 0.1% v/v TFA and collected in 1.5 ml microcentrifuge tubes. Peptides were then lyophilized by freeze drying (Virtis, SP Scientific, Warminster, PA). The peptides were resuspended in 100 μl distilled water and quantified using microBCA assay (Catalogue Number: 23235, Thermo Fischer Scientific) with BSA as the mass standard. Then, 10 μg of each sample (in a volume of 50 μl containing 100 mm TEAB) were differentially labeled by reductive dimethyl labeling using equal volumes (2 μl) of 4% light formaldehyde (CH_2_O), 4% medium formaldehyde (CD_2_O, 98% D) or heavy formaldehyde (^13^CD_2_O, 99% ^13^C, 98% D) and 0.6 m Sodium cyanoborohydride (NaCNBH_3_, for light and medium label) or Sodium cyanoborodeuteride (NaCNBD_3_, 96% D, for heavy label) added in sequence. The peptide solutions were incubated on an Eppendorf Thermomixer (Eppendorf South Pacific Pty. Ltd., Macquarie Park, NSW, Australia) at room temperature for 1 h. After quenching with 8 μl of 1% v/v ammonium hydroxide followed by 8 μl of neat formaldehyde, dimethyl-labeled peptides were mixed up in equal volumes before LC-MS/MS analysis.

Samples were analyzed by liquid chromatography-nano electrospray ionization-tandem mass spectrometry (LC-nESI-MS/MS) using Orbitrap Lumos mass spectrometer (Thermo Fisher Scientific) fitted with nanoflow reversed-phase-HPLC (Ultimate 3000 RSLC, Dionex, Thermo Fisher Scientific). The nano-LC system was equipped with an Acclaim Pepmap nano-trap column (Dionex - C18, 100 Å, 75 μm × 2 cm) and an Acclaim Pepmap RSLC analytical column (Dionex - C18, 100 Å, 75 μm × 50 cm, Thermo Fisher Scientific). For each LC-MS/MS experiment, 1 μg (whole proteome) or L (0.135 μg peptide) of the peptide mix was loaded onto the enrichment (trap) column at an isocratic flow of 5 μl/min of 3% CH_3_CN containing 0.1% formic acid for 6 min before the enrichment column is switched in-line with the analytical column. The eluents used for the LC were 5% DMSO/0.1% v/v formic acid (solvent A) and 100% CH_3_CN/5% DMSO/0.1% formic acid v/v. The gradient used was 3% B to 20% B for 95 min, 20% B to 40% B in 10 min, 40% B to 80% B in 5 min and maintained at 80% B for the final 5 min before equilibration for 10 min at 3% B prior to the next analysis.

The mass spectrometer was operated in positive-ionization mode with spray voltage set at 1.9 kV and source temperature at 275 °C. Lockmass of 401.92272 from DMSO was used. The mass spectrometer was operated in the data-dependent acquisition mode MS spectra scanning from *m*/*z* 400–1500 at 120,000 resolution with AGC target of 5e^5^. The “top speed” acquisition method mode (3 s cycle time) on the most intense precursor was used whereby peptide ions with charge states ≥2–5 were isolated with isolation window of 1.6 *m*/*z* and fragmented with high energy collision (HCD) mode with stepped collision energy of 30 ± 5%. Fragment ion spectra were acquired in Orbitrap at 15,000 resolution. Dynamic exclusion was activated for 30s.

##### Proteomic Data Analysis

For GFP-immunoprecipitated samples, raw MS data were analyzed using Proteome Discoverer (version 2.3.0.81; Thermo Fisher Scientific) with the Mascot search engine (Matrix Science version 2.4.1). Data were filtered against the SwissProt *Mus Musculus* database (version 2016_07; 16794 proteins) combined with common contaminant proteins. GFP sequence (UniProt ID: P42212) was also added to the database. For protein identification, the search was conducted with 20 ppm MS tolerance, and 0.8 Da MS/MS tolerance. The enzyme specificity was set as trypsin. The maximum number of missed cleavage sites permitted was two, and the minimum peptide length required was six. The following modifications were allowed: Oxidation (M), Acetylation (Protein N-term), Dimethylation (K), Dimethylation (N-Term), Dimethylation: 2H(4) (K), Dimethylation 2H(4) (N-term), 2H(6)13C(2) Dimethylation (K), 2H(6)13C(2) Dimethylation (N-term) (Variable); Carbamidomethyl (C) (Fixed). The false discovery rate (FDR) was calculated by the Percolator node in Proteome Discoverer v 2.3.0.81. Peptide identifications were accepted with a FDR threshold value of 0.01. Protein identifications were accepted with a FDR threshold of 0.05. Proteins were filtered for those identified by at least two peptides, one of which was unique, in all three replicates. The common contaminant, Keratin, was excluded from the data set.

Peptide quantitation was performed in Proteome Discoverer v.2.3.0.81 using the precursor ion quantifier node. Dimethyl labeled peptide pairs (between two comparisons of light, medium or heavy) were established with a 2 ppm mass precision, and a signal to noise threshold of 3. A retention time tolerance of isotope pattern multiplets was set to 0.8 min. Three single peak or missing channels were allowed for peptide identification. The protein abundance in each replicate was calculated by summation of the unique peptide abundances that were used for quantitation (light, medium and-or heavy dimethyl derivatives). Missing quantitation values were replaced with a constant (zero-filling). The peptide group abundance and protein abundance values were normalized to account for sample loading. In brief, the total peptide abundances for each sample was calculated and the maximum sum for all files was determined. The normalization factor was the factor of the sum of the sample and the maximum sum in all files. After calculating the normalization factors, the Peptide and Protein Quantifier node normalized peptide group abundances and protein abundances by dividing abundances with the normalization factor over all samples.

The normalized protein abundances were imported into Perseus software (v 1.6.5.0). Protein abundances were transformed to log_2_ scale. The samples were then grouped according to the replicates. For pairwise comparison of proteomes and determination of significant differences in protein abundances Welch's *t* test based on permutation-based FDR statistics was then applied (250 permutations; FDR = 0.01; S0 = 1). This, and all other t-tests below, are justified on the basis the proteomics abundance data is normally distributed.

For whole proteome data analysis, the conditions were like those above, but with the following differences: Raw MS data were analyzed using Proteome Discoverer (version 2.2.0.388; Thermo Fisher Scientific). For protein identification, the search was conducted with 20 ppm MS tolerance, and 0.6 Da MS/MS tolerance. The maximum number of missed cleavage sites permitted was three. Additional variable modifications for mono- and dimethylation of arginine were included. Peptide quantitation was performed in Proteome Discoverer v.2.2.0.388, and the retention time tolerance of isotope pattern multiplets was set to 0.6 min. After grouping samples, proteins with at least four valid values across all groups were retained for the subsequent analysis. Missing values were imputed from distribution of all other log_2_-transformed protein values from that sample, using the default settings in Perseus (1.8 standard deviation downshift, 0.3 mean downshift). For pairwise comparison of proteomes and determination of significant differences in protein abundances, a two-sample Students *t* test based on permutation-based FDR statistics was applied (250 permutations; FDR = 0.05; S0 = 0.1).

##### Dual Fluorescence Translation Stall Assay

Genes were synthesized to produce the dual-fluorescence translation stall reporter as described previously (30), except we used mCherry as the red fluorescent protein. DPR constructs or Httex1 with different polyQ expansions (25Q, 72Q and 97Q) were cloned to replace the linker region using PstI and BamHI restriction sites. Frame shifts were corrected using standard PCR-based strategies and were validated by sequencing. Dual fluorescence reporter plasmids were transfected into cells using Lipofectamine 2000 (Life Technologies) according to manufacturer guidelines. Two days after transfection, cells were harvested in PBS containing SYTOX Blue dead cell stain (S34857, Thermo Fischer Scientific) to exclude dead cells from subsequent analysis. Cellular fluorescence of 100,000 events per sample was analyzed on a LSRFortessa X-20 flow cytometer (BD Biosciences) using the 488-nm laser and 530/30 filter for GFP and the 561 nm laser and 610/20 bandpass filter for mCherry. Subsequent analysis of flow cytometry data was done using FlowJo software (v10.5.3) and graphs were analyzed in GraphPad Prism 7.05.

##### Flow Cytometry Analysis of G- and F-actin

F- and G-actin levels in Neuro2a cells were measured as described (31). Briefly, Neuro2a cells seeded into 12-well plates were harvested 48 h following transfection of different GFP-tagged DPRs. Cells were treated with 2 μm cytochalasin-D in 0.1% DMSO/PBS for 1 h for the positive control. Cells incubated with 0.1% DMSO for 1 h were used as negative control (untreated cells). Cells were fixed with 4% paraformaldehyde for 15 min, then permeabilized with 0.2% Triton X-100 for 5 min. After washing in PBS, cells were blocked with 1% BSA in 0.1% v/v Triton X-100/PBS solution for 15 min, and then incubated in the dark at room temperature for 30 min with Alexa Flour 594 deoxyribonuclease1 (DNase1) conjugate (10 μg ml^−1^, Invitrogen Molecular Probes, D12372) for G-actin detection and Alexaflour-405 Phalloidin (1:1000, Invitrogen Molecular Probes, A30104) for F-actin detection. After thorough washing with 1× PBS, fluorescence was measured using blue (BV421), green (FITC) and red (PE-Texas Red) channels on a FACSCanto flow cytometer (BD Bioscience). A total of 1 × 10^4^ cells were analyzed per acquisition. Unstained cells were used to set the baseline. G- and F-actin contents were determined from the respective fluorescence and the ratio of F/G calculated from the mean values as determined with FlowJo software.

##### Confocal Microscopy for F-actin Analysis

Cells were grown in 8-well ibidi culture chambers (Sarstedt, Nümbrecht, Germany). Cells were washed with PBS once before being fixed with 4% paraformaldehyde for 15 min at room temperature, then washed with PBS 3 times and permeabilized in 0.2% w/v Triton X-100/PBS for 5 min. The cells were then blocked with 1% w/v BSA in 0.1% w/v Triton X-100/PBS for 15 min at room temperature. F-actin was stained with Alexa Fluor 594 phalloidin (1:1000, Invitrogen, A12381) for 30 min and with Hoechst 33342 (1:200, Thermo Fisher Scientific) for nuclei staining for 30 min in the dark. Cells were imaged on a Leica SP5 confocal microscope (Leica Microsystems, Macquarie Park, Australia) using HCX PL APO CS 40× or 63× oil-immersion objective (NA 1.4) at room temperature. Fluorescence intensity profiles of phalloidin in >50 cells expressing GFP-tagged DPRs and untransfected cells were analyzed using ImageJ software.

##### Bioinformatic Analysis

Protein interaction networks were generated using Cytoscape 3.7.1 (32) built-in String (v11.0) (33) with a minimum required interaction score setting of 0.9 for interactome data or 0.7 for whole proteome data. Protein abundance changes were used as node color attributes and the node size reflected the Welch's *t* test *p* value. Nodes were manually re-arranged based on GO terms. Adobe Illustrator was used to annotate GO terms on the protein interaction network.

Cytoscape 3.7.1 plugin BiNGO was used to perform GO term analyses performed on proteins significantly enriched in the long *versus* short Arg-rich DPRs immuneprecipitates. Overrepresented categories were chosen after Benjamini & Hochberg False Discovery Rate correction. Hypergeometric statistical test was used to ascertain the *p* value of GO term enrichment. The annotation plots show the GO terms comprising at least three proteins with Benjamini-Hochberg adjusted *p* < 0.05 and the log_2_ fold enrichment of their associated proteins were significantly different from zero as assessed by one-sample student *t* test.

A Python algorithm developed by Mitrea *et al.* (34) was employed in this study to identify proteins exhibiting multivalent arginine-rich motifs (R-motifs) with the sequence pattern, RX_n1_RX_n2_RX_n3_R, where *n*1, *n*3 ≤ 2 and *n*2 ≤ 20. This algorithm was applied to the DPR-interacting proteins as well as proteins up- or downregulated upon DPR expression. The background proteins observed in the whole proteome analysis and which were not deemed significantly affected in abundance by DPR expression was used as a control. For determining significant differences, Fisher exact test was used.

##### Statistical Analysis

Statistical parameters are reported in the figures and corresponding figure legends. All statistical analyses were performed with GraphPad Prism v 7.05 (Graphpad Software Inc., San Diego, CA).

## RESULTS

### 

#### 

##### Characterization of the Cell Model Expressing the DPRs

Expression constructs of the DPRs were synthesized using mixed codons and an ATG-start codon, designed to minimize the influence of RNA-mediated effects from the repeat sequences. 10× and 101× repeat lengths of the toxic poly-PR and poly-GR, as well as the less toxic polyGA and polyAP, were prepared.

**Fig. 1.**
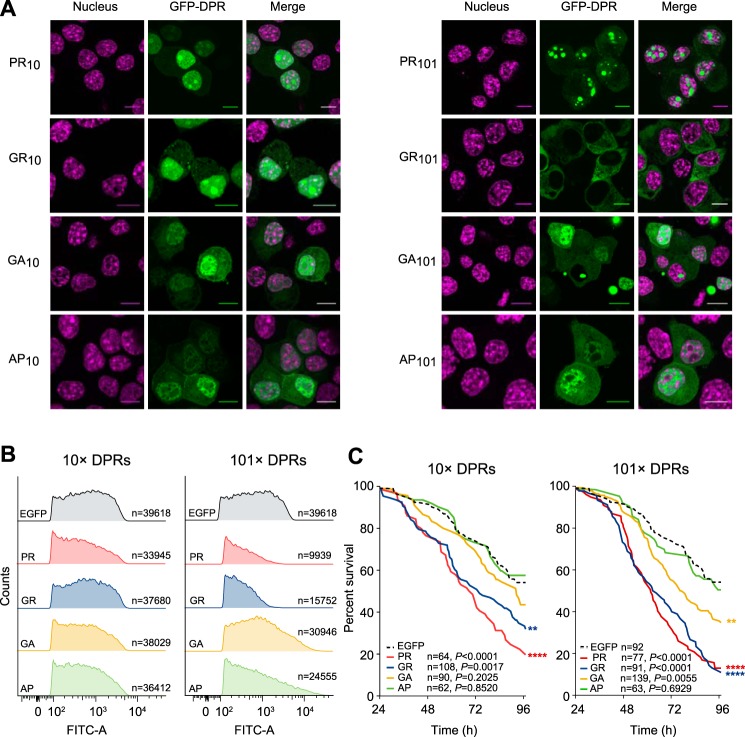
**DPR toxicity and cellular localization.** *A*, Confocal micrographs of Neuro2a cells 48 h after transfection with GFP-tagged DPRs. The nucleus was stained with Hoechst 33258. Scale bars represent 10 μm. *B*, Expression level of the DPRs as assessed by flow cytometry, 48 h following transfection. FITC-A channel tracks the GFP fluorescence. Shown are histograms of cell populations. *C*, Kaplan-Meier survival analysis of Neuro-2a transfected with the GFP-tagged DPRs. Cells were tracked from 24 h post-transfection. *p* values correspond to log-rank (Mantel-Cox) test of each sample *versus* EGFP control.

These constructs displayed patterns of localization and toxicity in the mouse neuroblastoma cell line Neuro2a similar to that described previously in other models (15–18, 35, 36) and observed *in vivo* for cases of MND with *C9ORF72* mutation (5, 14, 37, 38). This included a predominately nuclear punctate pattern of localization for PR_10_ and PR_101_ with residual expression in the cytosol (Fig. 1*A*). The longer repeat length accentuated the localization into the nuclear foci. GR_10_ also formed nuclear foci and appeared almost identical to PR_10_, however GR_101_ was excluded from the nucleus and had a mostly diffuse cytoplasmic distribution. By contrast, GA_10_, AP_10_ and AP_101_ were slightly enriched in the nucleus without forming distinct puncta. Similarly, GA_101_ was enriched in the nucleus but also formed large cytosolic (and less commonly nuclear) inclusions in many cells. All DPR constructs expressed at levels comparable to that of GFP except for PR_101_ and GR_101_ that have very weak expression levels; about 30% lower than that of either GFP or other long DPRs (Fig. 1*B*). These constructs also had a lower transfection efficiency (supplemental Fig. S1). According to cell survival rates when expressing the DPRs, the poly-PR and poly-GR constructs were most toxic and this was true in both 10× and 101× lengths (Fig. 1*C*). In contrast, poly-GA was only toxic in 101× lengths and the other DPRS were not toxic.

##### Arginine-rich DPRs Attracts Promiscuous Proteome Interactions

**Fig. 2.**
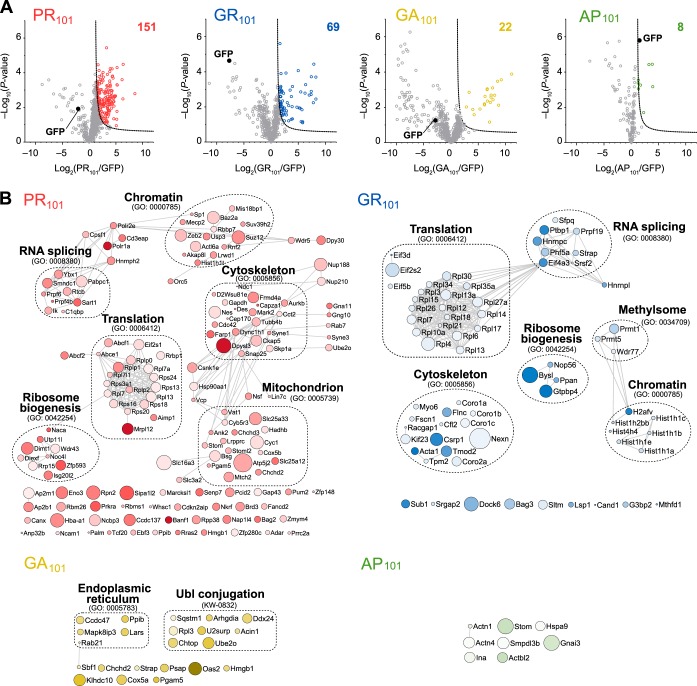
**Interactome analysis of the DPR_101_ variants.** *A*, Volcano plots of each DPR_101_-GFP *versus* GFP-only control from quantitative proteomic analysis of GFP-Trap immunoprecipitates of DPR_101_-GFP transfected in Neuro2a cells harvested 48h after transfection. Significant binders (shown in colored circles) were classified with False Discovery Rate of ≤ 0.01 (dotted lines). The number of interactors is indicated. *n* = 4 biological replicates for PR and AP and *n* = 3 biological replicates for GR and GA. *B*, STRING (v10) interaction maps for proteins significantly enriched with confidence set at 0.9 (highest stringency). Circle sizes are proportional to −log_10_ (*p* value). The color intensity is proportional to the log_2_ (fold change). Selected significantly enriched GO terms (GOCC, GOPB, and UniProt keywords) are displayed.

With the model system established, we next sought to investigate how the toxic 101× length DPRs (poly-GR, poly-PR and poly-GA) interacted with the proteome. To do this we transiently transfected cells with GFP or the 101× DPRs fused to GFP and then captured proteins that bound to the DPRs by immunoprecipitation with GFP trap. Proteins were assessed by quantitative proteomics by comparing the pulldowns to GFP-only transfected cells, with protein levels normalized to protein mass recovered from the immunoprecipitation. Under these conditions, GFP levels were anticipated to differ in the pulldown because of different expression levels (Fig 1*B*). Indeed, the amount of GFP appeared heavily enriched in the GFP-only control for the toxic DPRs (PR_101_, GR_101_ and to a lesser extent GA_101_) (Fig 2*A*). Yet, despite the larger amount of GFP coming from the control GFP-only transfected cells (which would enrich for nonspecific interactors to GFP), we observed many proteins strongly enriched to the Arg-rich DPRs and comparatively few for GA_101_ and AP_101_ (Fig 2*A*; supplemental Table S2). The result suggested two conclusions. One was that the Arg appears to mediate promiscuous binding to the proteome and the second was that these interactions are responsible for mitigating toxicity. The Arg-rich DPRs enriched for proteins involved in ribosome biogenesis and RNA splicing machinery, which is consistent with prior findings (18, 36, 39, 40). However, we also found additional novel interactions with proteins involved in ribosome-translation, cytoskeleton and chromatin machineries (Fig 2*B*). GR_101_ also enriched specifically for methylosome proteins and PR_101_ with mitochondrial proteins. GA_101_, which is the only DPR that formed large cytosolic inclusions, was enriched for a very distinct proteome. These interactions may be indicative of a distinct set of molecular mechanisms involved in its more modest toxicity.

##### Arginine-rich DPRs Lead to Ribosome Stalling

**Fig. 3.**
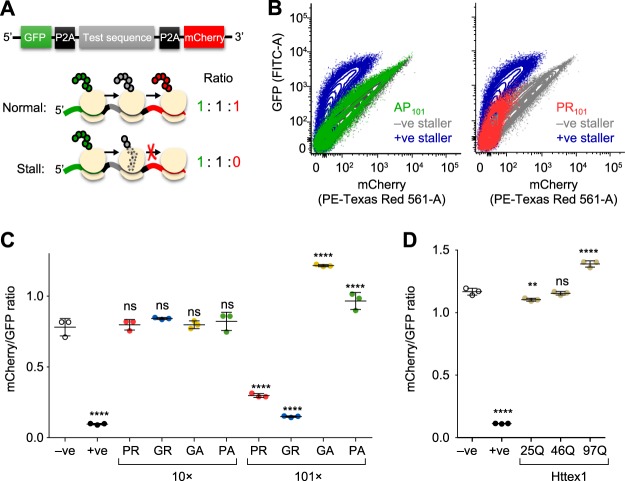
**Long Arg-rich DPRs stall ribosomes during translation.** *A*, Schematic of the reporter construct design. The P2A sequence causes the ribosome to skip the formation of a peptide bond but otherwise continue translation elongation uninterrupted. Complete translation of the cassette from one ribosome will generate three independent proteins (GFP, test protein, and mCherry). However, should the ribosome stall during synthesis (such as through the previously established positive stall reporter sequence with poly-lysine (K20; +ve sequence. The −ve sequence is stall reporter sequence without lysine residues. *B*, Flow cytograms of Neuro2a cells 48 h after transfection with the indicated test sequences inserted into the reporter. *C*, Median mCherry:GFP ratios calculated from transfected cells population (from 100,000 analyzed cells). Error bars indicate standard deviations from three independent transfections and flow cytometry measurements. *p* values determined for one-way ANOVA and Dunnett's post hoc test using the −ve as the control. ****, *p* < 0.0001; ***, *p* < 0.001; ns, *p* > 0.05. *D*, The same assay (and statistical tests) using Huntington exon 1 (Httex1) transfected in HEK293 cells with the indicated polyglutamine (polyQ) lengths.

It was previously reported that Arg-rich DPRs can cause translational suppression although a mechanism that remains undetermined (21). Our proteomics data revealed the ATP-binding cassette sub-family E member 1 (ABCE1) was enriched in the PR_101_ interactome. ABCE1 is involved in translation termination ribosomal recycling (41), which led us to wonder whether synthesis of the Arg-rich DPRs impairs translation. To examine this possibility, we employed a previously established assay to measure the ability of a protein sequence to stall or delay protein synthesis rates (30). This assay involves a cassette containing two fluorescent reporters on each side of the peptide sequence to be tested for stalling (GFP at the N terminus and mCherry at the C terminus) (Fig 3*A*). Each construct is encoded in frame without stop codons. However, the test sequence is flanked by viral P2A sequences, which causes the ribosome to skip the formation of a peptide bond but otherwise continue translation elongation uninterrupted. This means that complete translation of the cassette from one ribosome will generate three independent proteins (GFP, test protein, and mCherry) in an equal stoichiometry. However, should the ribosome stall during synthesis, mCherry is produced at lower stoichiometries than the GFP (Fig 3*A*).

Compared with previously validated FLAG-tagged stalling reporters that either contain 21 AAA codons (which therefore encodes poly-lysine) to stall, or no AAA codons to allow read-through (30), GR_101_ and PR_101_ constructs induced marked stalling (Fig 3*B*). This was DPR-length-dependent in that the 10× repeats showed no stalling (Fig 3*C*). In addition, the AP_101_ and GA_101_ did not lead to stalling (Fig 3*C*). Indeed, there was a significantly increased ratio of mCherry to GFP (Fig 3*C*). A likely explanation for this increased ratio comes from intermolecular fluorescence resonance energy transfer (FRET) arising from low rates of translational readthrough of the stall construct and self-association of these read-through protein products. This was more evident in another control construct of mutant Huntington exon 1 (Httex1), which when containing a polyglutamine (polyQ) expansion above 36 glutamines causes Huntington Disease and becomes highly aggregation prone (42, 43). The expanded polyQ forms of Httex1 did not cause stalling (Fig *3**D*). However, increased pathological lengths of polyglutamine increased the ratio of GFP to mCherry. Microscopic images confirmed the presence of GFP and mCherry in aggregates in cells expressing the GA_101_ and Httex1 reporters (supplemental Fig. S2).

##### Arginine-rich DPRs Alter the State of the Actin Cytoskeleton

**Fig. 4.**
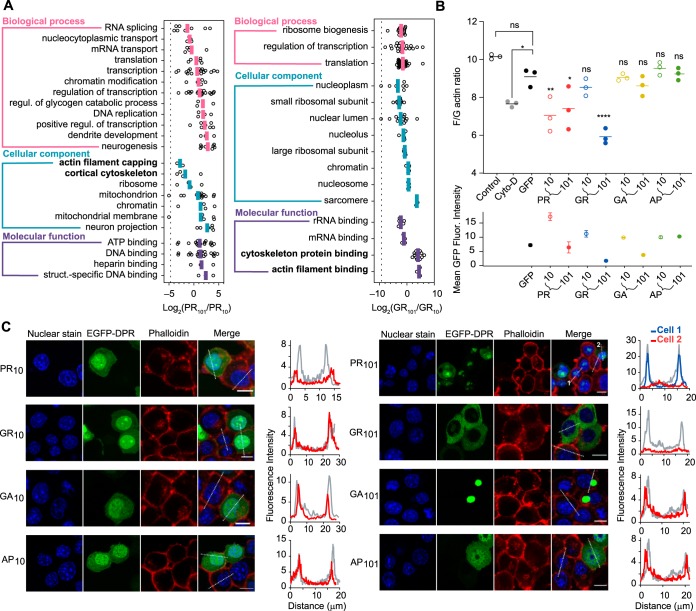
**Arg-content of the DPRs and subcellular location influence actin cytoskeleton assembly.** *A*, Gene Ontology (GO) enrichment analysis of proteins significantly associated with the long and short Arg-rich DPRs in the immunoprecipitation. GO terms are shown that contain at least three proteins, and where the log_2_ ratios of DPR length-enrichment values (DPR_101_/DPR_10_), shown as open circles, were deemed different to zero by a Student *t* test and Benjamini-Hochberg-adjusted *p* < 0.05. The colored lines indicate the means of the ratios. The dashed line represents the abundance ratio of GFP, which therefore provides an estimate of the bias of each DPR in the immunoprecipitation. GO terms related to cytoskeleton are shown in bold. *B*, Flow cytometry analyses of population ratios (1 × 10^4^ Neuro2a cells) of F-actin stain (stained with Phalloidin) and G-actin stain (stained with DNase I) 48 h after transfection with the GFP-tagged DPRs. Cyto-D cells represent 1 h Cytochalasin-D treatment in untransfected cells; Control indicates vehicle control treatment (0.1% DMSO) for Cyto-D sample. The lower graph shows the corresponding mean GFP fluorescence intensities of the samples in the upper graph. Data represent mean ± S.D., *n* = 3 with *p* values determined from a one-way ANOVA and Bonferroni's multiple comparisons post hoc-test with the GFP-alone sample as the control: *, *p* < 0.05; **, *p* < 0.01; ****, *p* < 0.0001; ns, *p* > 0.05. *C*, Intensity distance trajectories of F-actin (stained with Phalloidin) in Neuro2a cells transfected with GFP-tagged DPRs and nuclei stained with Hoechst 33258. Trajectories are shown on the cells as dotted white lines. Comparisons are shown in the graphs of representative cells (from over 50 measured cells) expressing GFP-tagged DPRs (red or blue lines) and untransfected cells in adjacent cells (gray lines). Scale Bars, 10 μm.

We next sought to examine whether the interactomes of the Arg-rich DPRs were influence by the length of the repeat sequence (Fig 4*A*). For this analysis, we measured the relative enrichment of the interacting proteins (101× *versus* 10×) in each gene ontology (GO) term significantly associated with the Arg-rich DPRs of both lengths. (The enrichment data of 10× DPRs compared with GFP-control are shown in supplemental Fig. S3). Almost all GO terms were significantly enriched to the longer Arg-rich DPRs and all were enriched once the relative abundance of the DPRs in the immunoprecipitations was considered (Fig 4*A*). These data therefore suggest arginine content generally mediates the binding, which may arise through increased charge per molecule (*i.e.* arg valency) or greater aggregation capacity. We also saw enrichment patterns consistent with different cellular localizations of 10× and 101× PR constructs (as shown in Fig. 1). In particular, the PR_101_ DPR revealed a substantial enrichment of proteins in GO terms relevant to nucleus localization (including DNA replication, positive regulation of transcription) which was consistent with the enhanced localization of PR_101_ to nucleolar substructures compared with PR_10_ (Fig 4*A*). Although the actin-related GO terms were enriched also for the Arg-rich DPRs, the enrichment correlated with the DPR localization in the cytosol where most actin is expected to reside. Namely, there was a lesser enrichment for PR_101_ compared with PR_10_, in accordance with the shift from more diffuse nuclear and cytoplasmic localization into the nucleolar substructures. GR_101_ was also excluded from the nucleus compared with GR_10_, and this was reflected in the greater enrichment of GR_101_ with actin-related GO terms.

Next we examined whether the enrichment with actin GO terms was indicative of changes in the actin cytoskeleton. The Arg-rich DPRs had a significant impact on the formation of filamentous (F) actin compared with the other DPRs and GFP-alone control using a flow cytometry protocol for measuring filamentous (F) and globular (G) actin ratios (Fig 4*B*). There was no apparent colocalization of actin to the DPRs, certainly not to punctate structures of the DPRs (Fig 4*C*). However, it was clear that F actin was reduced in individual cells expressing the Arg-rich DPRs (Fig 4*C*). Therefore, the results collectively suggested that the Arg-rich DPRs, either directly or indirectly, leads to destabilization of machinery involved in actin filament assembly without binding to the actin filaments directly.

**Fig. 5.**
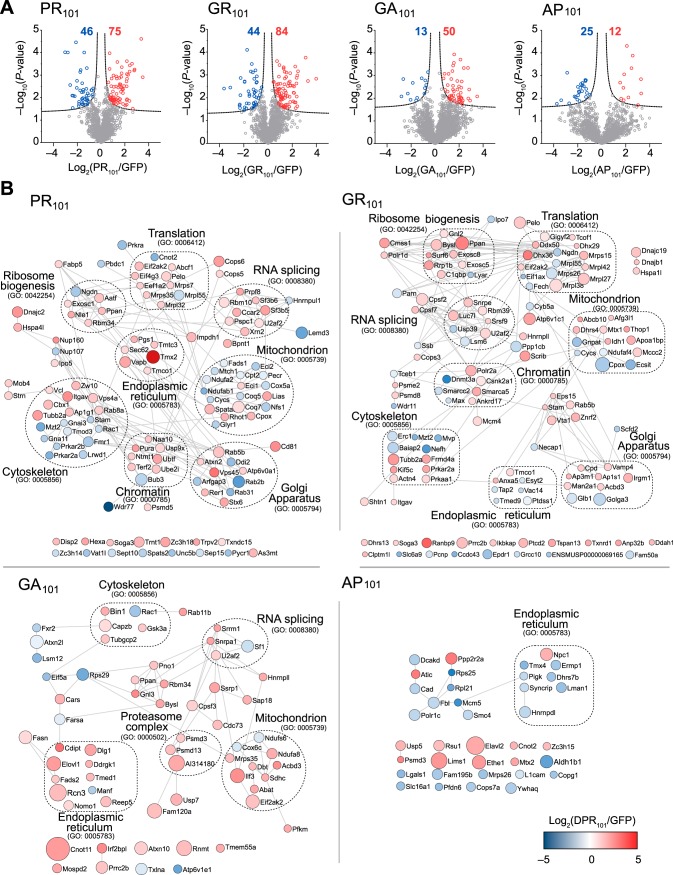
**Proteome abundance changes in response to DPR_101_ expression.** *A*, Shown are protein abundances in Neuro2a cells transfected with each GFP-DPR_101_ compared with GFP for 48 h, and sorted for matched GFP abundance by flow cytometry. *n* = 3 biological replicates. *p* values were determined with a two-sided Welch's t-Test. Significantly changed proteins (red and blue) were defined with FDR ≤ 0.01, S_0_ = 1 (dashed lines). The total number of proteins changed are indicated. *B*, Protein interaction networks for proteins significantly changed in abundance, color coded to enrichment using STRING (v10) in Cytoscape (v3.6) at medium confidence. Size of nodes scale proportionally to −log_10_ (*p* value). Selected significantly enriched GO terms (GOCC, GOPB, and UniProt keywords) are displayed.

Next, we examined how proteome abundances changed in cells expressing the 100× DPRs. Because the DPRs were toxic and had variable expression levels, we sorted transfected cells into similar levels of expression by virtue of GFP levels before analysis. Cell lysates were collected and quantitatively analyzed by a reductive dimethyl labeling proteome analysis approach (Fig 5*A*; supplemental Table S3). The poly-GR and poly-PR DPRs resulted in the most changes to the proteome in accordance with them having a more potent toxicity and promiscuous pattern of interactions. Poly-GR and poly-PR also had many overlapping GO terms annotated consistent with the Arg content driving a similar pathological consequence (Fig 5*B*). Conversely, the non-toxic poly-AP resulted in few changes to the proteome abundance and poly-GA had a distinct signature consistent with a different mechanism of toxicity.

##### Arginine-rich DPRs Lead to Altered Arginine Methylation Patterns

**Fig. 6.**
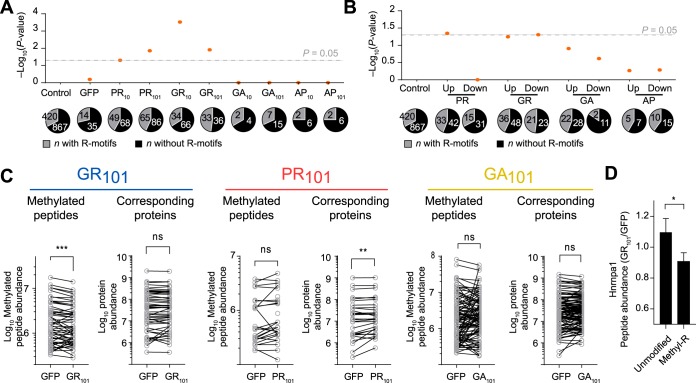
**Proteome hypomethylation upon GFP-GR_101_ expression.** *A*, Shown are *p* values of Fisher exact tests for enrichment of arginine motifs (34) in the DPR interactomes *versus* Control, which are the background proteins observed in the whole proteome analysis and which were not deemed significantly affected in abundance by DPR expression. Numbers of proteins are indicated. *B*, As for *A*, but for proteins seen significantly up-regulated and downregulated in total protein lysate compared with Control. *C*, Abundances of methylated peptides seen in the whole proteome, and the corresponding protein abundances from which they derive. The data is plotted as matched-pairs of peptides (or proteins) with differences evaluated by 2-tailed Wilcoxon signed-rank test. *p* values are coded as ns > 0.05; **, *p* < 0.01; ***, *p* < 0.001. The mean difference in abundances of matched pairs of methylated peptides (GR_101_ - GFP) is −399,018. *D*, Shown are the means ± S.E. of peptide abundance ratios of Hnrnpa1 from the PR101 and GFP samples. Shown are unmodified (*n* = 24) and arg-methylated peptides (*n* = 5). Significance of difference was assessed with an unpaired *t* test with Welch's correction. *p* value is coded as *, *p* < 0.05.

Arginine methylation has been reported to be abnormal in patients with *C9ORF72* mutations, including the presence of arginine-dimethylated enriched inclusions (44, 45). Arginine residues are commonly methylated to regulate biological activity of many cellular processes and are important in histones which are enriched GO terms in our data sets. In addition, abnormal histone methylation has previously been reported in a mouse model of PR_50_ (46). Furthermore, prior work has suggested that arginine methylase PRMT5 is important in regulating stress granule function in *C9ORF72* models of disease and methylates ALS-gene risk factor FUS (44). Here, GR_101_ interactome was found to be significantly enriched for 3 proteins in the Methylosome GO term (GO:0034709) including WDR77 and arginine methylases PRMT1 and PRMT5 (Fig 2*B*). PRMT1 appears particularly important, accounting for 85% of the methylation activity in mammalian cells (47). Analysis of the interactome data showed that the Arg-rich DPRs were significantly enriching for other arginine-enriched proteins that are common substrates for methylases (Fig 6*A* and 6*B*). This led us to hypothesize that the Arg-rich DPRs interfere with endogenous arginine methylation activity, that links to the mechanisms of ALS toxicity. To test this hypothesis, we examined the 101× DPRs affected proteome levels and corresponding levels of arginine methylation (supplemental Table S4). Because of our reductive dimethylation proteomics workflow, we could only observe a minor fraction of possible arginine methylation patterns that could exist. However, enough information was obtained to reveal that GR_101_ leads to a significantly lower level of arginine methylation relative to the GFP only control (Fig 6*C*; supplemental Table S3). These same proteins did not show a significant change in abundance (Fig 6*C*). Examination of these peptides revealed that many come from Hnrnp family proteins including Hnrnpa1, Hnrnpab and Hnrnph1 (supplemental Table S3). Hnrnpa1 showed a statistically significant reduction in Arg-methylation in the GR_101_ treatment (Fig 6*D*). Hnrnp family proteins are well-known substrates of PRMT family proteins (48). Mutations in Hnrnpa1 also cause ALS (49). Altogether, these data suggest the possibility that the Arg-rich DPRs act as substrate sinks of arginine methylases that therefore results in a broader deficiency in arginine methylase modification of endogenous proteins.

## DISCUSSION

Here we show that the Arg in the Arg-rich DPRs promotes widespread interactions with the proteome relative to the other less toxic DPRs. These interactions center on various hubs of cellular activity including translation, ribosome biogenesis, chromatin, mitochondria, cytoskeleton, RNA splicing and the methylosome. The effects may be driven by the valency of arginine as well as changes in the cellular localization, and potentially aggregation state, of the different lengths of DPRs. In contrast the inert and non-toxic AP DPRs showed few interactions. GA also showed relative few interactors and unmasked a distinct interactome, consistent with a different mechanism of toxicity to the potently toxic Arg-rich DPRs.

Overall our data are consistent with the Arg-rich DPRs manifesting toxicity through multiple mechanisms. One mechanism appears to arise by the arg-rich sequences causing translation to stall. Our results suggested that lengths longer than at least 10 repeats are needed to induce stalling. Given that the ribosome exit tunnel holds around 33 amino acids and is lined in negative charges, a plausible explanation for stalling is that DPR lengths approaching 16–17 repeat lengths would be sufficient to fill this cavity volume and lock the peptide in place by electrostatic interactions. Previously it was suggested that a canonical polyadenylate tail on mRNA used to stall ribosomes is translated to lysine and that the poly-lysine sequence is recognized as aberrant by ribosomes and results in translation repression (50). Additional experiments using repeating sequences of Lys and Arg in proteins both slowed translation, which supports the mechanism of electrostatic interactions jamming the emergent poly-basic chain in the negatively charged ribosome exit tunnel (51). Interestingly, antimicrobial peptides enriched for PR-containing motifs have been demonstrated to bind to the ribosomal exit tunnel and inhibit bacterial protein synthesis (52). This raises the possibility that emergent DPRs can also re-enter and plug the exit tunnel through electrostatic interactions. Further support for this additional mechanism comes from *in vitro* translation assays showing that poly-PR and poly-GR peptides formed insoluble complexes with mRNA, restricted the access of translation factors to mRNA, and blocked protein translation (23). This study showed that poly-PR and poly-GR inhibit protein translation by binding to the translational complex and ribosomal proteins, leading to neurotoxicity (23).

Our data also suggested that the Arg-rich DPRs impede assembly of the actin cytoskeleton. Recently it was observed that promoting actin filament assembly in cell models of ALS can alleviate defects in nuclear-cytoplasmic transport defects (53) which supports the conclusion that destabilization of the actin cytoskeleton is pertinent to disease pathomechanisms. We observed a significant reduction in F-actin in cells with Arg-rich DPRs. Whether this effect is a consequence of other cellular effects, such as hypomethylation or ribosome stalling is unclear. Interestingly it has been reported that arginine methylation of an arginine methylase (PRMT2) regulates the activity of actin nucleator protein Cobl, which suggests a possible role for arginine methylation defects being an upstream mediator of effects on the actin cytoskeleton (54). In addition, it is thought that dysregulation of actin is a key process in ALS (55). Of note is that mutations in profilin-1 protein, which mediates the conversion of G-actin to F-actin, is linked to ALS (56). Other cytoskeletal genes have also been linked to ALS including *TUBA4A* and *DCTN1* and *KIF5A* (57, 58).

Another mechanism of toxicity attributable to the Arg-rich DPRs was through a broader hypomethylation of the proteome. Previous studies have shown that PRMT1 colocalizes with GR and PR in a *Drosophila* model and that knockdown of PRMT family members enhanced toxicity (59). It was also found that *C9ORF72*-related brain samples had abundant methylated inclusions (59). Thus, the data raises the possibility that the Arg-rich motifs attract and alter the endogenous methylation activity leading to pathological outcomes. The substrate of PRMT family proteins contain glycine- and arginine-rich (GAR) sequences that include multiple arginines in RGG or RXR contexts, which bear resemblance to the Arg-rich DPRs (60). It follows that many of the key pathways seen in our data set are affected by altered arginine methylase activity - including proteins that are methylated for functional regulation such as histones, proteins involved in mRNA splicing, and ribosomes (61, 62).

Also of note is that other genes that when mutated are risk factors for ALS have activity regulated by arginine methylation and show abnormal methylation patterns in disease. In particular, FUS has been reported to interact with PRMT1 and PRMT8 and undergo asymmetric dimethylation in cultured cells (63). Importantly, PRMT1 and PRMT8 localized to mutant FUS-positive inclusion bodies in ALS (63). It has also been reported that arginine methylation modulates the nuclear import of FUS and inclusions in ALS-FUS patients contain methylated FUS (64). We observed the hypomethylation of Hnrnpa1 caused by the arg-rich DPRs, which indicates a possible link to ALS arising from distinct gene mutations. Hnrnpa1 as well as the Arg-rich DPRs and other ALS-associated proteins are known to form molecular condensates by phase separation (39, 58). Arginine methylation of Hnrnpa1 reduces its ability to phase separate suggesting that an imbalance in molecular condensate mechanisms contributes to the pathogenic response (65). PR DPRs can also promote the aggregation of ALS-related proteins containing prion-like domains, that are involved in mediating phase separation into molecular condensates (39). Hence our data indicate a possible convergence of multipronged mechanisms involving methylation, phase separation and cytoskeleton as important contributors to the toxicity of the Arg-rich DPRs.

## DATA AVAILABILITY

The mass spectrometry proteomics data have been deposited in the ProteomeXchange Consortium database via the PRIDE (66) partner repository program with the data set identifier PXD015177 (for interactome data, http://proteomecentral.proteomexchange.org/cgi/GetDataset?ID=PXD015177) and PXD015180 (for the whole proteome data, http://proteomecentral.proteomexchange.org/cgi/GetDataset?ID=PXD015180). The information of the identified proteins are provided as the supplemental data. The authors declare that they have no conflicts of interest with the contents of this article.

## Supplementary Material

Supplemental Information

Table S2

Table S3

Table S4

## Footnotes

^1^ The abbreviations used are:

MND: motor neuron disease
DPR: dipeptide repeat
FTD: frontotemporal degeneration
Httex1: Huntington exon 1
polyQ: polyglutamine
GO: gene ontology
LC-nESI-MS/MS: liquid chromatography-nano electrospray ionization–tandem mass spectrometry
FDR: false discovery rate.

## References

[1] Majounie E., Renton A. E., Mok K., Dopper E. G., Waite A., Rollinson S., Chio A., Restagno G., Nicolaou N., Simon-Sanchez J., van Swieten J. C., Abramzon Y., Johnson J. O., Sendtner M., Pamphlett R., Orrell R. W., Mead S., Sidle K. C., Houlden H., Rohrer J. D., Morrison K. E., Pall H., Talbot K., Ansorge O., Chromosome A. L. S. F. T. D. C., French research network on, F. F. A., Consortium I., Hernandez D. G., Arepalli S., Sabatelli M., Mora G., Corbo M., Giannini F., Calvo A., Englund E., Borghero G., Floris G. L., Remes A. M., Laaksovirta H., McCluskey L., Trojanowski J. Q., Van Deerlin V. M., Schellenberg G. D., Nalls M. A., Drory V. E., Lu C. S., Yeh T. H., Ishiura H., Takahashi Y., Tsuji S., Le Ber I., Brice A., Drepper C., Williams N., Kirby J., Shaw P., Hardy J., Tienari P. J., Heutink P., Morris H. R., Pickering-Brown S., and Traynor B. J. (2012) Frequency of the C9orf72 hexanucleotide repeat expansion in patients with amyotrophic lateral sclerosis and frontotemporal dementia: a cross-sectional study. Lancet Neurol. 11, 323–330 22406228 10.1016/S1474-4422(12)70043-1 PMC3322422

[2] DeJesus-Hernandez M., Mackenzie I. R., Boeve B. F., Boxer A. L., Baker M., Rutherford N. J., Nicholson A. M., Finch N. A., Flynn H., Adamson J., Kouri N., Wojtas A., Sengdy P., Hsiung G. Y., Karydas A., Seeley W. W., Josephs K. A., Coppola G., Geschwind D. H., Wszolek Z. K., Feldman H., Knopman D. S., Petersen R. C., Miller B. L., Dickson D. W., Boylan K. B., Graff-Radford N. R., and Rademakers R. (2011) Expanded GGGGCC hexanucleotide repeat in noncoding region of C9ORF72 causes chromosome 9p-linked FTD and ALS. Neuron 72, 245–256 21944778 10.1016/j.neuron.2011.09.011 PMC3202986

[3] Renton A. E., Majounie E., Waite A., Simon-Sanchez J., Rollinson S., Gibbs J. R., Schymick J. C., Laaksovirta H., van Swieten J. C., Myllykangas L., Kalimo H., Paetau A., Abramzon Y., Remes A. M., Kaganovich A., Scholz S. W., Duckworth J., Ding J., Harmer D. W., Hernandez D. G., Johnson J. O., Mok K., Ryten M., Trabzuni D., Guerreiro R. J., Orrell R. W., Neal J., Murray A., Pearson J., Jansen I. E., Sondervan D., Seelaar H., Blake D., Young K., Halliwell N., Callister J. B., Toulson G., Richardson A., Gerhard A., Snowden J., Mann D., Neary D., Nalls M. A., Peuralinna T., Jansson L., Isoviita V. M., Kaivorinne A. L., Holtta-Vuori M., Ikonen E., Sulkava R., Benatar M., Wuu J., Chio A., Restagno G., Borghero G., Sabatelli M., Consortium I., Heckerman D., Rogaeva E., Zinman L., Rothstein J. D., Sendtner M., Drepper C., Eichler E. E., Alkan C., Abdullaev Z., Pack S. D., Dutra A., Pak E., Hardy J., Singleton A., Williams N. M., Heutink P., Pickering-Brown S., Morris H. R., Tienari P. J., and Traynor B. J. (2011) A hexanucleotide repeat expansion in C9ORF72 is the cause of chromosome 9p21-linked ALS-FTD. Neuron 72, 257–268 21944779 10.1016/j.neuron.2011.09.010 PMC3200438

[4] Gomez-Tortosa E., Gallego J., Guerrero-Lopez R., Marcos A., Gil-Neciga E., Sainz M. J., Diaz A., Franco-Macias E., Trujillo-Tiebas M. J., Ayuso C., and Perez-Perez J. (2013) C9ORF72 hexanucleotide expansions of 20–22 repeats are associated with frontotemporal deterioration. Neurology 80, 366–370 23284068 10.1212/WNL.0b013e31827f08ea

[5] Gendron T. F., Bieniek K. F., Zhang Y. J., Jansen-West K., Ash P. E., Caulfield T., Daughrity L., Dunmore J. H., Castanedes-Casey M., Chew J., Cosio D. M., van Blitterswijk M., Lee W. C., Rademakers R., Boylan K. B., Dickson D. W., and Petrucelli L. (2013) Antisense transcripts of the expanded C9ORF72 hexanucleotide repeat form nuclear RNA foci and undergo repeat-associated non-ATG translation in c9FTD/ALS. Acta Neuropathol. 126, 829–844 24129584 10.1007/s00401-013-1192-8 PMC3830741

[6] Cooper-Knock J., Walsh M. J., Higginbottom A., Robin Highley J., Dickman M. J., Edbauer D., Ince P. G., Wharton S. B., Wilson S. A., Kirby J., Hautbergue G. M., and Shaw P. J. (2014) Sequestration of multiple RNA recognition motif-containing proteins by C9orf72 repeat expansions. Brain 137, 2040–2051 24866055 10.1093/brain/awu120 PMC4065024

[7] Sareen D., O'Rourke J. G., Meera P., Muhammad A. K., Grant S., Simpkinson M., Bell S., Carmona S., Ornelas L., Sahabian A., Gendron T., Petrucelli L., Baughn M., Ravits J., Harms M. B., Rigo F., Bennett C. F., Otis T. S., Svendsen C. N., and Baloh R. H. (2013) Targeting RNA foci in iPSC-derived motor neurons from ALS patients with a C9ORF72 repeat expansion. Sci. Transl. Med. 5, 208ra149 10.1126/scitranslmed.3007529 PMC4090945 24154603

[8] Mori K., Lammich S., Mackenzie I. R., Forne I., Zilow S., Kretzschmar H., Edbauer D., Janssens J., Kleinberger G., Cruts M., Herms J., Neumann M., Van Broeckhoven C., Arzberger T., and Haass C. (2013) hnRNP A3 binds to GGGGCC repeats and is a constituent of p62-positive/TDP43-negative inclusions in the hippocampus of patients with C9orf72 mutations. Acta Neuropathol. 125, 413–423 23381195 10.1007/s00401-013-1088-7

[9] Donnelly C. J., Zhang P. W., Pham J. T., Haeusler A. R., Mistry N. A., Vidensky S., Daley E. L., Poth E. M., Hoover B., Fines D. M., Maragakis N., Tienari P. J., Petrucelli L., Traynor B. J., Wang J., Rigo F., Bennett C. F., Blackshaw S., Sattler R., and Rothstein J. D. (2013) RNA toxicity from the ALS/FTD C9ORF72 expansion is mitigated by antisense intervention. Neuron 80, 415–428 24139042 10.1016/j.neuron.2013.10.015 PMC4098943

[10] Lee Y. B., Chen H. J., Peres J. N., Gomez-Deza J., Attig J., Stalekar M., Troakes C., Nishimura A. L., Scotter E. L., Vance C., Adachi Y., Sardone V., Miller J. W., Smith B. N., Gallo J. M., Ule J., Hirth F., Rogelj B., Houart C., and Shaw C. E. (2013) Hexanucleotide repeats in ALS/FTD form length-dependent RNA foci, sequester RNA binding proteins, and are neurotoxic. Cell Rep. 5, 1178–1186 24290757 10.1016/j.celrep.2013.10.049 PMC3898469

[11] Zu T., Gibbens B., Doty N. S., Gomes-Pereira M., Huguet A., Stone M. D., Margolis J., Peterson M., Markowski T. W., Ingram M. A., Nan Z., Forster C., Low W. C., Schoser B., Somia N. V., Clark H. B., Schmechel S., Bitterman P. B., Gourdon G., Swanson M. S., Moseley M., and Ranum L. P. (2011) Non-ATG-initiated translation directed by microsatellite expansions. Proc. Natl. Acad. Sci. U.S.A. 108, 260–265 21173221 10.1073/pnas.1013343108 PMC3017129

[12] Ash P. E., Bieniek K. F., Gendron T. F., Caulfield T., Lin W. L., Dejesus-Hernandez M., van Blitterswijk M. M., Jansen-West K., Paul J. W. 3rd, Rademakers R., Boylan K. B., Dickson D. W., and Petrucelli L. (2013) Unconventional translation of C9ORF72 GGGGCC expansion generates insoluble polypeptides specific to c9FTD/ALS. Neuron 77, 639–646 23415312 10.1016/j.neuron.2013.02.004 PMC3593233

[13] Mori K., Arzberger T., Grasser F. A., Gijselinck I., May S., Rentzsch K., Weng S. M., Schludi M. H., van der Zee J., Cruts M., Van Broeckhoven C., Kremmer E., Kretzschmar H. A., Haass C., and Edbauer D. (2013) Bidirectional transcripts of the expanded C9orf72 hexanucleotide repeat are translated into aggregating dipeptide repeat proteins. Acta Neuropathol. 126, 881–893 24132570 10.1007/s00401-013-1189-3

[14] Zu T., Liu Y., Banez-Coronel M., Reid T., Pletnikova O., Lewis J., Miller T. M., Harms M. B., Falchook A. E., Subramony S. H., Ostrow L. W., Rothstein J. D., Troncoso J. C., and Ranum L. P. (2013) RAN proteins and RNA foci from antisense transcripts in C9ORF72 ALS and frontotemporal dementia. Proc. Natl. Acad. Sci. U.S.A. 110, E4968–E4977 24248382 10.1073/pnas.1315438110 PMC3870665

[15] Mizielinska S., Gronke S., Niccoli T., Ridler C. E., Clayton E. L., Devoy A., Moens T., Norona F. E., Woollacott I. O. C., Pietrzyk J., Cleverley K., Nicoll A. J., Pickering-Brown S., Dols J., Cabecinha M., Hendrich O., Fratta P., Fisher E. M. C., Partridge L., and Isaacs A. M. (2014) C9orf72 repeat expansions cause neurodegeneration in Drosophila through arginine-rich proteins. Science 345, 1192–1194 25103406 10.1126/science.1256800 PMC4944841

[16] Wen X., Tan W., Westergard T., Krishnamurthy K., Markandaiah S. S., Shi Y., Lin S., Shneider N. A., Monaghan J., Pandey U. B., Pasinelli P., Ichida J. K., and Trotti D. (2014) Antisense proline-arginine RAN dipeptides linked to C9ORF72-ALS/FTD form toxic nuclear aggregates that initiate in vitro and in vivo neuronal death. Neuron 84, 1213–1225 25521377 10.1016/j.neuron.2014.12.010 PMC4632245

[17] May S., Hornburg D., Schludi M. H., Arzberger T., Rentzsch K., Schwenk B. M., Grasser F. A., Mori K., Kremmer E., Banzhaf-Strathmann J., Mann M., Meissner F., and Edbauer D. (2014) C9orf72 FTLD/ALS-associated Gly-Ala dipeptide repeat proteins cause neuronal toxicity and Unc119 sequestration. Acta Neuropathol. 128, 485–503 25120191 10.1007/s00401-014-1329-4 PMC4159571

[18] Lee K. H., Zhang P., Kim H. J., Mitrea D. M., Sarkar M., Freibaum B. D., Cika J., Coughlin M., Messing J., Molliex A., Maxwell B. A., Kim N. C., Temirov J., Moore J., Kolaitis R. M., Shaw T. I., Bai B., Peng J., Kriwacki R. W., and Taylor J. P. (2016) C9orf72 dipeptide repeats impair the assembly, dynamics, and function of membrane-less organelles. Cell 167, 774–788 e717 27768896 10.1016/j.cell.2016.10.002 PMC5079111

[19] Saberi S., Stauffer J. E., Jiang J., Garcia S. D., Taylor A. E., Schulte D., Ohkubo T., Schloffman C. L., Maldonado M., Baughn M., Rodriguez M. J., Pizzo D., Cleveland D., and Ravits J. (2018) Sense-encoded poly-GR dipeptide repeat proteins correlate to neurodegeneration and uniquely co-localize with TDP-43 in dendrites of repeat-expanded C9orf72 amyotrophic lateral sclerosis. Acta Neuropathol. 135, 459–474 29196813 10.1007/s00401-017-1793-8 PMC5935138

[20] Zhang Y. J., Gendron T. F., Ebbert M. T. W., O'Raw A. D., Yue M., Jansen-West K., Zhang X., Prudencio M., Chew J., Cook C. N., Daughrity L. M., Tong J., Song Y., Pickles S. R., Castanedes-Casey M., Kurti A., Rademakers R., Oskarsson B., Dickson D. W., Hu W., Gitler A. D., Fryer J. D., and Petrucelli L. (2018) Poly(GR) impairs protein translation and stress granule dynamics in C9orf72-associated frontotemporal dementia and amyotrophic lateral sclerosis. Nat. Med. 24, 1136–1142 29942091 10.1038/s41591-018-0071-1 PMC6520050

[21] Moens T. G., Niccoli T., Wilson K. M., Atilano M. L., Birsa N., Gittings L. M., Holbling B. V., Dyson M. C., Thoeng A., Neeves J., Glaria I., Yu L., Bussmann J., Storkebaum E., Pardo M., Choudhary J. S., Fratta P., Partridge L., and Isaacs A. M. (2019) C9orf72 arginine-rich dipeptide proteins interact with ribosomal proteins in vivo to induce a toxic translational arrest that is rescued by eIF1A. Acta Neuropathol. 137, 487–500 30604225 10.1007/s00401-018-1946-4 PMC6514073

[22] Kwon I., Xiang S., Kato M., Wu L., Theodoropoulos P., Wang T., Kim J., Yun J., Xie Y., and McKnight S. L. (2014) Poly-dipeptides encoded by the C9orf72 repeats bind nucleoli, impede RNA biogenesis, and kill cells. Science 345, 1139–1145 25081482 10.1126/science.1254917 PMC4459787

[23] Kanekura K., Yagi T., Cammack A. J., Mahadevan J., Kuroda M., Harms M. B., Miller T. M., and Urano F. (2016) Poly-dipeptides encoded by the C9ORF72 repeats block global protein translation. Hum. Mol. Genet. 25, 1803–1813 26931465 10.1093/hmg/ddw052 PMC4986334

[24] Tao Z., Wang H., Xia Q., Li K., Li K., Jiang X., Xu G., Wang G., and Ying Z. (2015) Nucleolar stress and impaired stress granule formation contribute to C9orf72 RAN translation-induced cytotoxicity. Hum. Mol. Genet. 24, 2426–2441 25575510 10.1093/hmg/ddv005

[25] Zhang K., Donnelly C. J., Haeusler A. R., Grima J. C., Machamer J. B., Steinwald P., Daley E. L., Miller S. J., Cunningham K. M., Vidensky S., Gupta S., Thomas M. A., Hong I., Chiu S. L., Huganir R. L., Ostrow L. W., Matunis M. J., Wang J., Sattler R., Lloyd T. E., and Rothstein J. D. (2015) The C9orf72 repeat expansion disrupts nucleocytoplasmic transport. Nature 525, 56–61 26308891 10.1038/nature14973 PMC4800742

[26] Freibaum B. D., and Taylor J. P. (2017) The role of dipeptide repeats in C9ORF72-related ALS-FTD. Front. Mol. Neurosci. 10, 35 28243191 10.3389/fnmol.2017.00035 PMC5303742

[27] Abramoff M. D., Magalhaes P. J., and Ram S. J. (2004) Image Processing with ImageJ. Biophotonics International. 11, 36–42

[28] Arrasate M., Mitra S., Schweitzer E. S., Segal M. R., and Finkbeiner S. (2004) Inclusion body formation reduces levels of mutant huntingtin and the risk of neuronal death. Nature 431, 805–810 15483602 10.1038/nature02998

[29] Strebel A., Harr T., Bachmann F., Wernli M., and Erb P. (2001) Green fluorescent protein as a novel tool to measure apoptosis and necrosis. Cytometry 43, 126–133 11169577 10.1002/1097-0320(20010201)43:2<126::aid-cyto1027>3.0.co;2-j

[30] Juszkiewicz S., and Hegde R. S. (2017) Initiation of quality control during poly(A) translation requires site-specific ribosome ubiquitination. Mol. Cell 65, 743–750 e744 28065601 10.1016/j.molcel.2016.11.039 PMC5316413

[31] Grosse R., Copeland J. W., Newsome T. P., Way M., and Treisman R. (2003) A role for VASP in RhoA-Diaphanous signalling to actin dynamics and SRF activity. EMBO J. 22, 3050–3061 12805219 10.1093/emboj/cdg287 PMC162139

[32] Shannon P., Markiel A., Ozier O., Baliga N. S., Wang J. T., Ramage D., Amin N., Schwikowski B., and Ideker T. (2003) Cytoscape: a software environment for integrated models of biomolecular interaction networks. Genome Res. 13, 2498–2504 14597658 10.1101/gr.1239303 PMC403769

[33] Szklarczyk D., Morris J. H., Cook H., Kuhn M., Wyder S., Simonovic M., Santos A., Doncheva N. T., Roth A., Bork P., Jensen L. J., and von Mering C. (2017) The STRING database in 2017: quality-controlled protein-protein association networks, made broadly accessible. Nucleic Acids Res. 45, D362–D368 27924014 10.1093/nar/gkw937 PMC5210637

[34] Mitrea D. M., Cika J. A., Guy C. S., Ban D., Banerjee P. R., Stanley C. B., Nourse A., Deniz A. A., and Kriwacki R. W. (2016) Nucleophosmin integrates within the nucleolus via multi-modal interactions with proteins displaying R-rich linear motifs and rRNA. Elife 5 10.7554/eLife.13571 PMC4786410 26836305

[35] Suzuki H., Shibagaki Y., Hattori S., and Matsuoka M. (2018) The proline-arginine repeat protein linked to C9-ALS/FTD causes neuronal toxicity by inhibiting the DEAD-box RNA helicase-mediated ribosome biogenesis. Cell Death Dis. 9, 975 30250194 10.1038/s41419-018-1028-5 PMC6155127

[36] Hartmann H., Hornburg D., Czuppa M., Bader J., Michaelsen M., Farny D., Arzberger T., Mann M., Meissner F., and Edbauer D. (2018) Proteomics and C9orf72 neuropathology identify ribosomes as poly-GR/PR interactors driving toxicity. Life Sci. Alliance 1, e201800070 30456350 10.26508/lsa.201800070 PMC6238541

[37] Mori K., Weng S. M., Arzberger T., May S., Rentzsch K., Kremmer E., Schmid B., Kretzschmar H. A., Cruts M., Van Broeckhoven C., Haass C., and Edbauer D. (2013) The C9orf72 GGGGCC repeat is translated into aggregating dipeptide-repeat proteins in FTLD/ALS. Science 339, 1335–1338 23393093 10.1126/science.1232927

[38] Schludi M. H., May S., Grasser F. A., Rentzsch K., Kremmer E., Kupper C., Klopstock T., German Consortium for Frontotemporal Lobar, D., Bavarian Brain Banking, A., Arzberger T., and Edbauer D. (2015) Distribution of dipeptide repeat proteins in cellular models and C9orf72 mutation cases suggests link to transcriptional silencing. Acta Neuropathol. 130, 537–555 26085200 10.1007/s00401-015-1450-z PMC4575390

[39] Boeynaems S., Bogaert E., Kovacs D., Konijnenberg A., Timmerman E., Volkov A., Guharoy M., De Decker M., Jaspers T., Ryan V. H., Janke A. M., Baatsen P., Vercruysse T., Kolaitis R. M., Daelemans D., Taylor J. P., Kedersha N., Anderson P., Impens F., Sobott F., Schymkowitz J., Rousseau F., Fawzi N. L., Robberecht W., Van Damme P., Tompa P., and Van Den Bosch L. (2017) Phase separation of C9orf72 dipeptide repeats perturbs stress granule dynamics. Mol. Cell 65, 1044–1055 e1045 28306503 10.1016/j.molcel.2017.02.013 PMC5364369

[40] Lin Y., Mori E., Kato M., Xiang S., Wu L., Kwon I., and McKnight S. L. (2016) Toxic PR poly-dipeptides encoded by the C9orf72 repeat expansion target LC domain polymers. Cell 167, 789–802 e712 27768897 10.1016/j.cell.2016.10.003 PMC5076566

[41] Pisarev A. V., Skabkin M. A., Pisareva V. P., Skabkina O. V., Rakotondrafara A. M., Hentze M. W., Hellen C. U., and Pestova T. V. (2010) The role of ABCE1 in eukaryotic posttermination ribosomal recycling. Mol. Cell 37, 196–210 20122402 10.1016/j.molcel.2009.12.034 PMC2951834

[42] Duyao M., Ambrose C., Myers R., Novelletto A., Persichetti F., Frontali M., Folstein S., Ross C., Franz M., Abbott M., and et al (1993) Trinucleotide repeat length instability and age of onset in Huntington's disease. Nat. Genet. 4, 387–392 8401587 10.1038/ng0893-387

[43] Scherzinger E., Sittler A., Schweiger K., Heiser V., Lurz R., Hasenbank R., Bates G. P., Lehrach H., and Wanker E. E. (1999) Self-assembly of polyglutamine-containing huntingtin fragments into amyloid-like fibrils: implications for Huntington's disease pathology. Proc. Natl. Acad. Sci. U.S.A. 96, 4604–4609 10200309 10.1073/pnas.96.8.4604 PMC16379

[44] Chitiprolu M., Jagow C., Tremblay V., Bondy-Chorney E., Paris G., Savard A., Palidwor G., Barry F. A., Zinman L., Keith J., Rogaeva E., Robertson J., Lavallee-Adam M., Woulfe J., Couture J. F., Cote J., and Gibbings D. (2018) A complex of C9ORF72 and p62 uses arginine methylation to eliminate stress granules by autophagy. Nat. Commun. 9, 2794 30022074 10.1038/s41467-018-05273-7 PMC6052026

[45] Sakae N., Bieniek K. F., Zhang Y. J., Ross K., Gendron T. F., Murray M. E., Rademakers R., Petrucelli L., and Dickson D. W. (2018) Poly-GR dipeptide repeat polymers correlate with neurodegeneration and Clinicopathological subtypes in C9ORF72-related brain disease. Acta Neuropathol. Commun. 6, 63 30029693 10.1186/s40478-018-0564-7 PMC6054740

[46] Zhang Y. J., Guo L., Gonzales P. K., Gendron T. F., Wu Y., Jansen-West K., O'Raw A. D., Pickles S. R., Prudencio M., Carlomagno Y., Gachechiladze M. A., Ludwig C., Tian R., Chew J., DeTure M., Lin W. L., Tong J., Daughrity L. M., Yue M., Song Y., Andersen J. W., Castanedes-Casey M., Kurti A., Datta A., Antognetti G., McCampbell A., Rademakers R., Oskarsson B., Dickson D. W., Kampmann M., Ward M. E., Fryer J. D., Link C. D., Shorter J., and Petrucelli L. (2019) Heterochromatin anomalies and double-stranded RNA accumulation underlie C9orf72 poly(PR) toxicity. Science 363, pii: eaav2606 10.1126/science.aav2606 PMC6524780 30765536

[47] Tang J., Frankel A., Cook R. J., Kim S., Paik W. K., Williams K. R., Clarke S., and Herschman H. R. (2000) PRMT1 is the predominant type I protein arginine methyltransferase in mammalian cells. J. Biol. Chem. 275, 7723–7730 10713084 10.1074/jbc.275.11.7723

[48] Dreyfuss G., Matunis M. J., Pinol-Roma S., and Burd C. G. (1993) hnRNP proteins and the biogenesis of mRNA. Annu. Rev. Biochem. 62, 289–321 8352591 10.1146/annurev.bi.62.070193.001445

[49] Kim H. J., Kim N. C., Wang Y. D., Scarborough E. A., Moore J., Diaz Z., MacLea K. S., Freibaum B., Li S., Molliex A., Kanagaraj A. P., Carter R., Boylan K. B., Wojtas A. M., Rademakers R., Pinkus J. L., Greenberg S. A., Trojanowski J. Q., Traynor B. J., Smith B. N., Topp S., Gkazi A. S., Miller J., Shaw C. E., Kottlors M., Kirschner J., Pestronk A., Li Y. R., Ford A. F., Gitler A. D., Benatar M., King O. D., Kimonis V. E., Ross E. D., Weihl C. C., Shorter J., and Taylor J. P. (2013) Mutations in prion-like domains in hnRNPA2B1 and hnRNPA1 cause multisystem proteinopathy and ALS. Nature 495, 467–473 23455423 10.1038/nature11922 PMC3756911

[50] Ito-Harashima S., Kuroha K., Tatematsu T., and Inada T. (2007) Translation of the poly(A) tail plays crucial roles in nonstop mRNA surveillance via translation repression and protein destabilization by proteasome in yeast. Genes Dev. 21, 519–524 17344413 10.1101/gad.1490207 PMC1820893

[51] Lu J., and Deutsch C. (2008) Electrostatics in the ribosomal tunnel modulate chain elongation rates. J. Mol. Biol. 384, 73–86 18822297 10.1016/j.jmb.2008.08.089 PMC2655213

[52] Gagnon M. G., Roy R. N., Lomakin I. B., Florin T., Mankin A. S., and Steitz T. A. (2016) Structures of proline-rich peptides bound to the ribosome reveal a common mechanism of protein synthesis inhibition. Nucleic Acids Res. 44, 2439–2450 26809677 10.1093/nar/gkw018 PMC4797290

[53] Giampetruzzi A., Danielson E. W., Gumina V., Jeon M., Boopathy S., Brown R. H., Ratti A., Landers J. E., and Fallini C. (2019) Modulation of actin polymerization affects nucleocytoplasmic transport in multiple forms of amyotrophic lateral sclerosis. Nat. Commun. 10, 3827 31444357 10.1038/s41467-019-11837-y PMC6707192

[54] Hou W., Nemitz S., Schopper S., Nielsen M. L., Kessels M. M., and Qualmann B. (2018) Arginine methylation by PRMT2 controls the functions of the actin nucleator Cobl. Dev. Cell 45, 262–275 e268 29689199 10.1016/j.devcel.2018.03.007

[55] Hensel N., and Claus P. (2018) The actin cytoskeleton in SMA and ALS: how does it contribute to motoneuron degeneration? Neuroscientist 24, 54–72 28459188 10.1177/1073858417705059

[56] Wu C. H., Fallini C., Ticozzi N., Keagle P. J., Sapp P. C., Piotrowska K., Lowe P., Koppers M., McKenna-Yasek D., Baron D. M., Kost J. E., Gonzalez-Perez P., Fox A. D., Adams J., Taroni F., Tiloca C., Leclerc A. L., Chafe S. C., Mangroo D., Moore M. J., Zitzewitz J. A., Xu Z. S., van den Berg L. H., Glass J. D., Siciliano G., Cirulli E. T., Goldstein D. B., Salachas F., Meininger V., Rossoll W., Ratti A., Gellera C., Bosco D. A., Bassell G. J., Silani V., Drory V. E., Brown R. H. Jr, and Landers J. E. (2012) Mutations in the profilin 1 gene cause familial amyotrophic lateral sclerosis. Nature 488, 499–503 22801503 10.1038/nature11280 PMC3575525

[57] Nicolas A., Kenna K. P., Renton A. E., Ticozzi N., Faghri F., Chia R., Dominov J. A., Kenna B. J., Nalls M. A., Keagle P., Rivera A. M., van Rheenen W., Murphy N. A., van Vugt J., Geiger J. T., Van der Spek R. A., Pliner H. A., Shankaracharya Smith B. N., Marangi G., Topp S. D., Abramzon Y., Gkazi A. S., Eicher J. D., Kenna A., Consortium I., Mora G., Calvo A., Mazzini L., Riva N., Mandrioli J., Caponnetto C., Battistini S., Volanti P., La Bella V., Conforti F. L., Borghero G., Messina S., Simone I. L., Trojsi F., Salvi F., Logullo F. O., D'Alfonso S., Corrado L., Capasso M., Ferrucci L., Genomic Translation for, A. L. S. C. C., Moreno C. A. M., Kamalakaran S., Goldstein D. B., Consortium A. L. S. S., Gitler A. D., Harris T., Myers R. M., Consortium N. A., Phatnani H., Musunuri R. L., Evani U. S., Abhyankar A., Zody M. C., Answer A. L. S. F., Kaye J., Finkbeiner S., Wyman S. K., LeNail A., Lima L., Fraenkel E., Svendsen C. N., Thompson L. M., Van Eyk J. E., Berry J. D., Miller T. M., Kolb S. J., Cudkowicz M., Baxi E., Clinical Research in, A. L. S., Related Disorders for Therapeutic Development, C., Benatar M., Taylor J. P., Rampersaud E., Wu G., Wuu J., Consortium S., Lauria G., Verde F., Fogh I., Tiloca C., Comi G. P., Soraru G., Cereda C., French A. L. S. C., Corcia P., Laaksovirta H., Myllykangas L., Jansson L., Valori M., Ealing J., Hamdalla H., Rollinson S., Pickering-Brown S., Orrell R. W., Sidle K. C., Malaspina A., Hardy J., Singleton A. B., Johnson J. O., Arepalli S., Sapp P. C., McKenna-Yasek D., Polak M., Asress S., Al-Sarraj S., King A., Troakes C., Vance C., de Belleroche J., Baas F., Ten Asbroek A., Munoz-Blanco J. L., Hernandez D. G., Ding J., Gibbs J. R., Scholz S. W., Floeter M. K., Campbell R. H., Landi F., Bowser R., Pulst S. M., Ravits J. M., MacGowan D. J. L., Kirby J., Pioro E. P., Pamphlett R., Broach J., Gerhard G., Dunckley T. L., Brady C. B., Kowall N. W., Troncoso J. C., Le Ber I., Mouzat K., Lumbroso S., Heiman-Patterson T. D., Kamel F., Van Den Bosch L., Baloh R. H., Strom T. M., Meitinger T., Shatunov A., Van Eijk K. R., de Carvalho M., Kooyman M., Middelkoop B., Moisse M., McLaughlin R. L., Van Es M. A., Weber M., Boylan K. B., Van Blitterswijk M., Rademakers R., Morrison K. E., Basak A. N., Mora J. S., Drory V. E., Shaw P. J., Turner M. R., Talbot K., Hardiman O., Williams K. L., Fifita J. A., Nicholson G. A., Blair I. P., Rouleau G. A., Esteban-Perez J., Garcia-Redondo A., Al-Chalabi A., Project Min E. A. L. S. S. C., Rogaeva E., Zinman L., Ostrow L. W., Maragakis N. J., Rothstein J. D., Simmons Z., Cooper-Knock J., Brice A., Goutman S. A., Feldman E. L., Gibson S. B., Taroni F., Ratti A., Gellera C., Van Damme P., Robberecht W., Fratta P., Sabatelli M., Lunetta C., Ludolph A. C., Andersen P. M., Weishaupt J. H., Camu W., Trojanowski J. Q., Van Deerlin V. M., Brown R. H. Jr., van den Berg L. H., Veldink J. H., Harms M. B., Glass J. D., Stone D. J., Tienari P., Silani V., Chio A., Shaw C. E., Traynor B. J., and Landers J. E. (2018) Genome-wide analyses identify KIF5A as a novel ALS gene. Neuron 97, 1268–1283.e1266 29566793 10.1016/j.neuron.2018.02.027 PMC5867896

[58] Taylor J. P., Brown R. H. Jr., and Cleveland D. W. (2016) Decoding ALS: from genes to mechanism. Nature 539, 197–206 27830784 10.1038/nature20413 PMC5585017

[59] Boeynaems S., Bogaert E., Michiels E., Gijselinck I., Sieben A., Jovicic A., De Baets G., Scheveneels W., Steyaert J., Cuijt I., Verstrepen K. J., Callaerts P., Rousseau F., Schymkowitz J., Cruts M., Van Broeckhoven C., Van Damme P., Gitler A. D., Robberecht W., and Van Den Bosch L. (2016) Drosophila screen connects nuclear transport genes to DPR pathology in c9ALS/FTD. Sci. Rep. 6, 20877 26869068 10.1038/srep20877 PMC4751451

[60] Blanc R. S., and Richard S. (2017) Arginine methylation: the coming of age. Mol. Cell 65, 8–24 28061334 10.1016/j.molcel.2016.11.003

[61] Yang Y., and Bedford M. T. (2013) Protein arginine methyltransferases and cancer. Nat. Rev. Cancer 13, 37–50 23235912 10.1038/nrc3409

[62] Chang F. N., Navickas I. J., Chang C. N., and Dancis B. M. (1976) Methylation of ribosomal proteins in HeLa cells. Arch. Biochem. Biophys. 172, 627–633 1259424 10.1016/0003-9861(76)90117-x

[63] Scaramuzzino C., Monaghan J., Milioto C., Lanson N. A. Jr., Maltare A., Aggarwal T., Casci I., Fackelmayer F. O., Pennuto M., and Pandey U. B. (2013) Protein arginine methyltransferase 1 and 8 interact with FUS to modify its sub-cellular distribution and toxicity in vitro and in vivo. PLoS ONE 8, e61576 23620769 10.1371/journal.pone.0061576 PMC3631215

[64] Dormann D., Madl T., Valori C. F., Bentmann E., Tahirovic S., Abou-Ajram C., Kremmer E., Ansorge O., Mackenzie I. R., Neumann M., and Haass C. (2012) Arginine methylation next to the PY-NLS modulates Transportin binding and nuclear import of FUS. EMBO J. 31, 4258–4275 22968170 10.1038/emboj.2012.261 PMC3501225

[65] Ryan V. H., Dignon G. L., Zerze G. H., Chabata C. V., Silva R., Conicella A. E., Amaya J., Burke K. A., Mittal J., and Fawzi N. L. (2018) Mechanistic view of hnRNPA2 low-complexity domain structure, interactions, and phase separation altered by mutation and arginine methylation. Mol. Cell 69, 465–479.e467 29358076 10.1016/j.molcel.2017.12.022 PMC5801700

[66] Vizcaino J. A., Csordas A., del-Toro N., Dianes J. A., Griss J., Lavidas I., Mayer G., Perez-Riverol Y., Reisinger F., Ternent T., Xu Q. W., Wang R., and Hermjakob H. (2016) 2016 update of the PRIDE database and its related tools. Nucleic Acids Res. 44, D447–D456 26527722 10.1093/nar/gkv1145 PMC4702828

