# Supplementary material for: Arginine in C9ORF72 Dipolypeptides Mediates Promiscuous Proteome Binding and Multiple Modes of Toxicity
Source: Mol Cell Proteomics. 2020 Feb 21;19(4):640–54. doi: 10.1074/mcp.RA119.001888 (PMC7124463; doi:10.1074/mcp.RA119.001888)
Supplement: Supplemental Information [file 157401_1_supp_477898_q5z1m4.pdf]

Supplemental materials for:

Arginine in *C9ORF72* polydipeptides mediates promiscuous proteome binding and multiple modes of toxicity.

Mona Radwan, Ching-Seng Ang, Angelique R. Ormsby, Dezerae Cox, James C. Daly, Gavin E. Reid, Danny M. Hatters

- **Table S1: Sequence of the open reading frames from the synthetic DPR expression constructs.**
- **Table S2: Protein interactors to 10× and 101× DPRs. Relates to Fig 2 & Fig S2.** *Separate file*
- **Table S3: Cellular abundances of proteins caused by 101× DPR expression. Relates to Fig 5.** *Separate file*
- **Table S4: Arginine methylated proteome quantitation by 101× DPR expression. Relates to Fig 6.** *Separate file*

- **Fig S1: Transfection efficiency of DPRs as fusions to GFP. Related to Fig 1.**
- **Fig S2: Expression of DPRs in the ribosome stall reporter constructs. Relates to Fig 3.**
- **Fig S3. Interactome analysis of the DPR_{10} variants. Relates to Fig 2.**

Table S1. Sequence of the open reading frames from the synthetic DPR expression constructs.

Construct	Sequence
**10x DPRs**	
GA_{10}	5’-GGCGCTGGCGCTGGGGCAGGCGCAGGGGCTGGCGCAGGCGCTGGGGCTGGGGCTGGGGCA-3’
GR_{10}	5’-GGCAGAGGAAGAGGCAGGGGACGCGGAAGGGGGAGAGGACGCGGCAGAGGCCGGGGAAGA-3’
AP_{10}	5’-GCACCAGCTCCAGCCCCTGCTCCTGCTCCCGCCCCAGCACCCGCCCCTGCCCCAGCCCCA-3’
PR_{10}	5’-CCCAGACCTAGACCTCGGCCTAGACCAAGACCCAGGCCAAGGCCACGGCCAAGACCTAGA-3’
**101x DPRs**	
GA_{101}	5’GCCGGCGCTGGCGCTGGGGCAGGCGCAGGGGCTGGCGCCGGGGCCGGGGCCGGCGCTGGCGCAGGGGCTGG<br>GGCTGGCGCAGGCGCTGGGGCAGGGGCTGGCGCTGGGGCTGGCGCAGGCGCAGGCGCTGGCGCTGGCGCAGGG<br>GCTGGCGCAGGCGCTGGGGCTGGCGCTGGGGCAGGGGCAGGGGCAGGGGCTGGGGCAGGCGCTGGCGCAGGC<br>GCAGGCGCAGGGGCAGGCGCTGGGGCTGGGGCTGGGGCTGGCGCAGGGGCCGGGGCCGGGGCAGGGGCAGGC<br>GCAGGCGCAGGGGCTGGGGCAGGGGCAGGCGCAGGGGCTGGCGCTGGCGCTGGCGCCGGGGCCGGGGCCGGC<br>GCAGGGGCTGGCGCTGGGGCAGGCGCTGGCGCAGGGGCAGGGGCAGGCGCTGGCGCTGGGGCAGGGGCTGGG<br>GCCGGGGCCGGCGCAGGCGCTGGGGCAGGCGCAGGCGCAGGCGCTGGGGCCGGGGCCGGGGCTGGCGCTGGC<br>GCTGGCGCAGGCGCTGGGGCTGGGGCAGGCGCCGGGGCCGGGGCCGGGGCAGGCGCTGGGGCTGGGGCTGGC<br>GCAGGGGCAGGCGCTGGGGCAGGCGCAGGGGCC-3’
GR_{101}	5’CGGGGCAGAGGCCGGGGAAGAGGCAGAGGACGCGGAAGGGGAAGGGGGAGAGGAAGAGGGCGGGGACGCG<br>GCCGGGGCCGCGGGCGGGGCCGGGGCCGGGGGCGCGGCCGGGGGCGGGGGCGCGGGCGCGGCCGGGGCCGG<br>GGCCGCGGGCGGGGGCGCGGCCGGGGCCGGGGGCGGGGGCGCGGGCGGGGGCGGGGAAGAGGCAGGGGCAG<br>AGGAAGAGGAAGAGGACGGGGGAGGGGCAGAGGAAGGGGACGCGGCAGAGGCAGGGGACGGGGACGCGGAA<br>GAGGAAGAGGCAGAGGCAGGGGGAGAGGACGGGGCAGGGGGAGGGGCAGGGGACGGGGGAGAGGACGCGG<br>ACGCGGACGCGGCAGGGGAAGAGGAAGGGGAAGGGGAAGAGGACGCGGCAGAGGACGGGGCAGAGGAAGAG<br>GCCGGGGAAGAGGACGCGGAAGGGGGAGAGGCCGCGGAAGAGGAAGGGGAAGAGGGCGGGGACGGGGAAGG<br>GGACGGGGACGGGGCAGAGGCAGAGGAAGGGGCAGGGGCAGGGGAAGGGGCAGAGGAAGAGGAAGGGGAAG<br>AGGCAGAGGAAGAGGACGCGGCAGAGGCCGCGGAAGAGGACGG-3’
AP_{101}	5’CCCGCCCCTGCCCCTGCTCCAGCTCCTGCACCAGCACCCGCTCCAGCCCCAGCCCCCGCACCCGCCCCTGCACCCG<br>CACCAGCACCAGCCCCTGCTCCAGCCCCCGCTCCTGCTCCTGCCCCAGCCCCAGCTCCCGCTCCAGCTCCTGCTCCCG<br>CTCCAGCCCCTGCACCAGCCCCTGCCCCCGCTCCCGCACCAGCTCCAGCACCAGCTCCCGCCCCTGCTCCAGCACCAG<br>CCCCAGCACCAGCACCAGCTCCCGCACCAGCCCCTGCCCCAGCTCCTGCTCCCGCCCCTGCTCCTGCCCCCGCACCCG<br>CACCCGCTCCCGCCCCAGCTCCAGCTCCTGCCCCTGCCCCCGCTCCAGCACCCGCCCCAGCTCCCGCTCCTGCACCCG<br>CTCCCGCTCCCGCACCCGCCCCAGCCCCTGCACCAGCTCCAGCTCCAGCTCCTGCTCCTGCACCAGCTCCCGCACCCG<br>CCCCAGCTCCAGCTCCCGCTCCAGCACCAGCCCCTGCCCCTGCCCCAGCCCCTGCCCCTGCACCAGCACCAGCACCC<br>GCTCCAGCACCCGCACCAGCCCCAGCACCCGCCCCTGCACCAGCTCCCGCCCCAGCCCCCGCTCCT-3’
PR_{101}	5’CGGCCCAGACCCCGGCCTAGACCAAGACCCAGACCAAGGCCAAGACCTCGGCCTCGCCCTAGGCCACGCCCTCGC<br>CCCAGACCTAGACCTAGGCCTCGGCCAAGACCTAGGCCCAGGCCTAGACCCCGCCCACGCCCTAGACCACGGCCAA<br>GGCCTAGACCTAGACCAAGGCCCAGGCCCAGACCAAGGCCTCGCCCCAGGCCAAGACCACGGCCAAGACCAAGAC<br>CACGCCCCAGACCCAGACCCAGACCTAGACCACGCCCTAGGCCAAGGCCTCGGCCACGGCCTCGGCCTAGACCCAG<br>GCCAAGACCTAGACCTCGGCCTAGACCACGGCCTCGCCCACGCCCAAGGCCAAGACCAAGACCTAGACCCCGGCCT<br>CGCCCAAGGCCCAGACCTCGCCCTAGACCTCGCCCAAGACCAAGGCCTAGACCTCGCCCACGGCCCAGACCTAGAC<br>CAAGACCACGGCCACGCCCTAGACCTAGGCCCAGACCTCGGCCCAGACCCAGACCACGGCCTAGACCTAGGCCAAG

	GCCACGCCCAAGGCCCAGGCCAAGGCCCAGACCAAGACCAAGACCCAGACCTCGGCCAAGGCCAAGGCCAAGACC<br>AAGG-3’

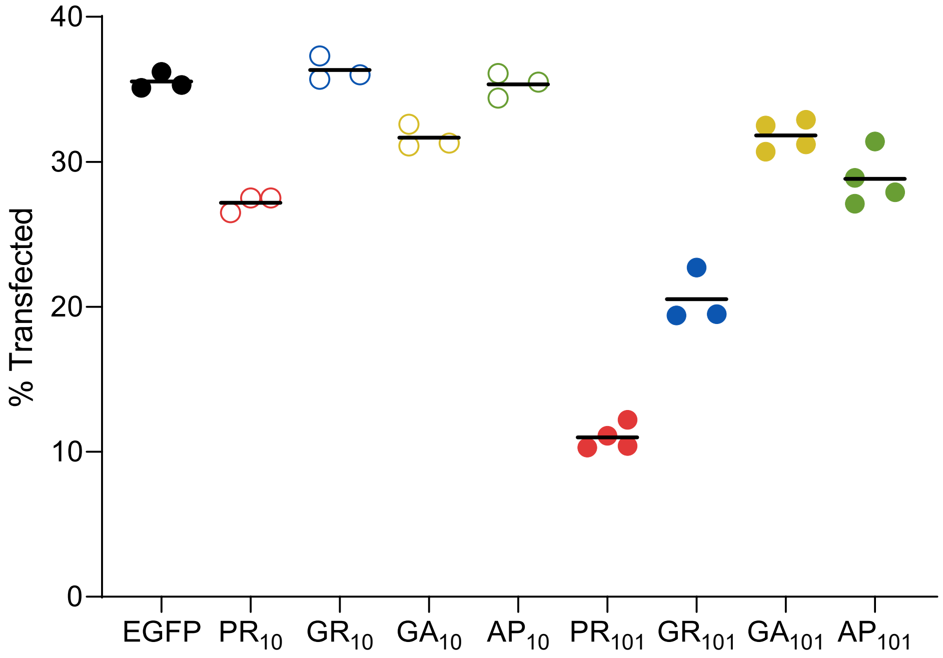

Fig S1: Transfection efficiency of DPRs as fusions to GFP. Related to Fig 1. Neuro2a cells were transfected with GFP-tagged DPRs (or EGFP alone) as labelled. Shown are percentages of cells in each sample with higher GFP fluorescence than background levels. The background levels were derived by reference to untransfected cells. Data were determined by flow cytometry analysis. Shown are biological replicates (circles) and means (bars).

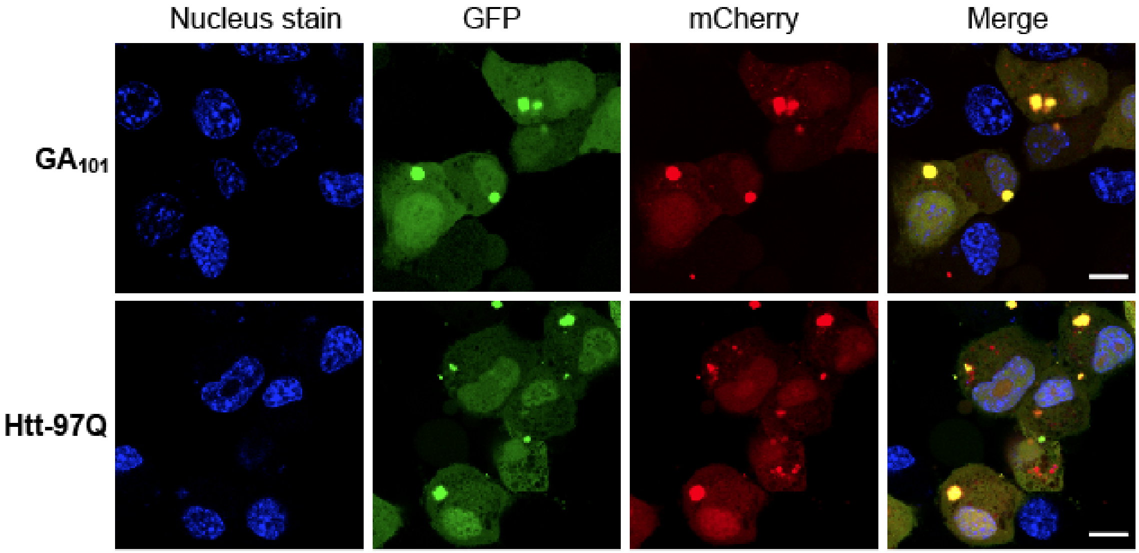

Fig S1: Expression of DPRs in the ribosome stall reporter constructs. Relates to Fig 3. Shown are confocal micrographs of Neuro2a cells overexpressing the reporter construct containing either GA_{101} (top panel) or Httex1 with 97Q repeats (bottom panel) fixed 48 hr post-transfection and stained with Hoechst 33258 (blue) to visualize nuclei. Scale bars represent 10 µm.

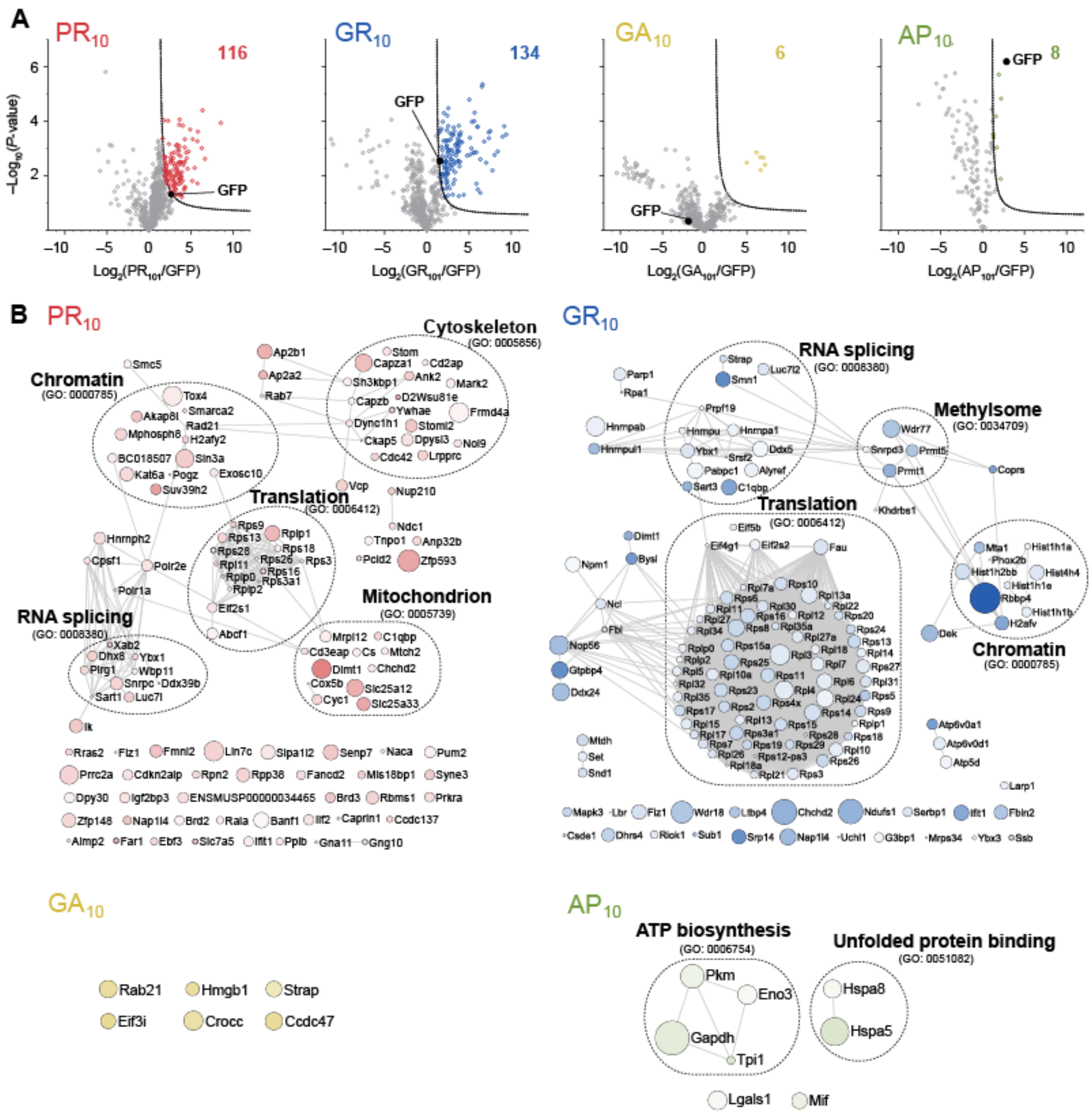

Fig S2. Interactome analysis of the DPR_{10} variants. Relates to Fig 2. A. Volcano Plots of each DPR_{10}-GFP versus GFP-only control of quantitative proteomics analysis of GFP-Trap immunoprecipitates of DPR_{10}-GFP transfected in Neuro2a cells harvested 48h after transfection. Significant binders (shown in colored circles) were classified with False Discovery Rate of ≤ 0.01 (dotted lines). The number of interactors are indicated. **B.** STRING (v10) interaction maps for proteins significantly enriched with confidence set at 0.9 (highest stringency). Circle sizes are proportional to $-\log_{10}$ (*P*-value). The color intensity is proportion to the log (fold change). Selected significantly enriched GO terms (GOCC, GOPB, and UniProt keywords) are displayed (with FDR cutoff *P* value < 0.05).
